# Co-Occurrence and Levels of Mycotoxins in Fish Feeds in Kenya

**DOI:** 10.3390/toxins12100627

**Published:** 2020-09-30

**Authors:** Evalyn Wanjiru Mwihia, Jan Ludvig Lyche, Paul Gichohi Mbuthia, Lada Ivanova, Silvio Uhlig, James K. Gathumbi, Joyce G. Maina, Eric Emali Eshitera, Gunnar Sundstøl Eriksen

**Affiliations:** 1 Department of Veterinary Pathology, Microbiology and Parasitology, Faculty of Veterinary Medicine and Surgery, Egerton University, P.O. Box 536, Egerton 20115, Kenya; 2 Department of Food Safety and Infectious Biology, Faculty of Veterinary Medicine, Norwegian University of Life Sciences (NMBU), P.O. Box 8146, 0454 Oslo, Norway; jan.l.lyche@nmbu.no; 3 Department of Pathology, Microbiology and Parasitology, Faculty of Veterinary Medicine, University of Nairobi, P.O. Box 29053, Kangemi 00625, Kenya; pgmbuthia@uonbi.ac.ke (P.G.M.); jkgathumbi@gmail.com (J.K.G.); 4 Toxinology Research Group, Norwegian Veterinary Institute, Ullevålsveien 68, Pb 750 Sentrum, 0106 Oslo, Norway; lada.ivanova@vetinst.no (L.I.); silvio.uhlig@vetinst.no (S.U.); 5 Department of Animal Production, Faculty of Veterinary Medicine, University of Nairobi, P.O. Box 29053, Kangemi 00625, Kenya; maina.joyce78@gmail.com; 6 Department of Animal Health and Production, School of Natural Resource and Animal Sciences, Maasai Mara University, P.O. Box 861, Narok 20500, Kenya; eshitera@mmarau.ac.ke

**Keywords:** mycotoxins, fish feeds, prevalence, co-occurrence, levels, Kenya, HPLC

## Abstract

This study determined the presence, levels and co-occurrence of mycotoxins in fish feeds in Kenya. Seventy-eight fish feeds and ingredients were sampled from fish farms and fish feed manufacturing plants and analysed for 40 mycotoxins using high-performance liquid chromatography-high resolution mass spectrometry. Twenty-nine (73%) mycotoxins were identified with 76 (97%) samples testing positive for mycotoxins presence. Mycotoxins with the highest prevalences were enniatin B (91%), deoxynivalenol (76%) and fumonisin B1 (54%) while those with the highest maximum levels were sterigmatocystin (<30.5–3517.1 µg/kg); moniliformin (<218.9–2583.4 µg/kg) and ergotamine (<29.3–1895.6 µg/kg). Mycotoxin co-occurrence was observed in 68 (87%) samples. Correlations were observed between the fumonisins; enniatins B and zearalenone and its metabolites. Fish dietary exposure estimates ranged between <0.16 and 43.38 µg/kg body weight per day. This study shows evidence of mycotoxin presence and co-occurrence in fish feeds and feed ingredients in Kenya. Fish exposure to these levels of mycotoxins over a long period of time may lead to adverse health effects due to their possible additive, synergistic or antagonist toxic effects. Measures to reduce fish feed mycotoxin contamination should be taken to avoid mycotoxicosis in fish and subsequently in humans and animals through residues.

## 1. Introduction

The term “mycotoxin” is derived from the Greek word, “mykes” meaning fungus (mould) and the Latin word “toxicum” meaning poison [1,2]; as such, mycotoxins refer to poisons produced by fungi. Specifically, mycotoxins are fungal secondary metabolites, which are toxic to vertebrates and other animal species in low concentrations [3]. They are produced mainly by fungi belonging to *Fusarium*, *Aspergillus*, *Claviceps* and *Penicillium* genera [4]. They are thought to be produced to assist the fungi to cope with oxidative stress and also as a defence mechanism against other organisms sharing the same trophic niche [4]. Over 300 mycotoxins have been identified [5], and the most common mycotoxins are: aflatoxins (AF); ochratoxin A (OTA); citrinin; patulin; deoxynivalenol (DON), T-2 toxin (T2) and HT-2 toxin (HT2); fumonisins (FUM) and zearalenone (ZEN) [6].

Exposure of human beings and other animals to mycotoxins is mainly through oral consumption of contaminated foods and feeds, however, skin contact and inhalation have also been identified as potential routes [3,7,8,9,10]. The severity of the toxic effect of mycotoxins on animals and human health (mycotoxicosis), is dependent on the type(s) of mycotoxin; the amount and duration of exposure and the age, health and sex of the exposed individual [2,3]. Mycotoxins have been reported to cause an array of adverse health effects ranging from morbidities, decreased production, poor quality of animal products to mortalities [10,11,12,13]. In fish, mycotoxins cause poor growth rates, carcinogenic effects, kidney damage, liver and gastrointestinal disturbances, reproductive disorders and suppression of the immune system depending on the type of mycotoxin, exposure period and doses [12,14,15,16,17].

Mycotoxins are commonly prevalent in a majority of feeds [18] and feed ingredients [13]. Co-occurrence may occur due to colonization by more than one fungal species which would produce different types of mycotoxins or colonization by one fungal species that produces more than one type of mycotoxin [19]. Exposure of animals and human beings to multiple mycotoxins may lead to additive, synergistic or antagonist toxic effects [20]. Published studies on the combined effects of multiple mycotoxins suggest possible greater toxic effects of the mixtures compared to individual mycotoxins [21].

Previous studies have confirmed the occurrence of various mycotoxins in different animal feeds and feed ingredients in Kenya [22,23,24,25,26,27,28,29,30,31,32,33,34]. However, of greatest emphasis have been aflatoxins which are highly prevalent in Kenya with the most recent severe epidemic that killed 125 people being reported in the year 2004 [35,36]. Even though other mycotoxins have been detected in Kenyan foods and feeds, the available data are scarce. Additionally, studies on co-occurrence of mycotoxins in fish feeds are not well documented and thus far, only one study carried out in the Lake Victoria region [33] exists. The aim of this study, therefore, was to determine the presence, levels and co-occurrence of various mycotoxins in fish feed in Kenya. The information herein may be used to assess the risk of mycotoxicoses in fish and by association, in man and other animals. It can also be used to justify the implementation of mycotoxin management strategies.

## 2. Results

### 2.1. Samples Composition

**Figure 1:**
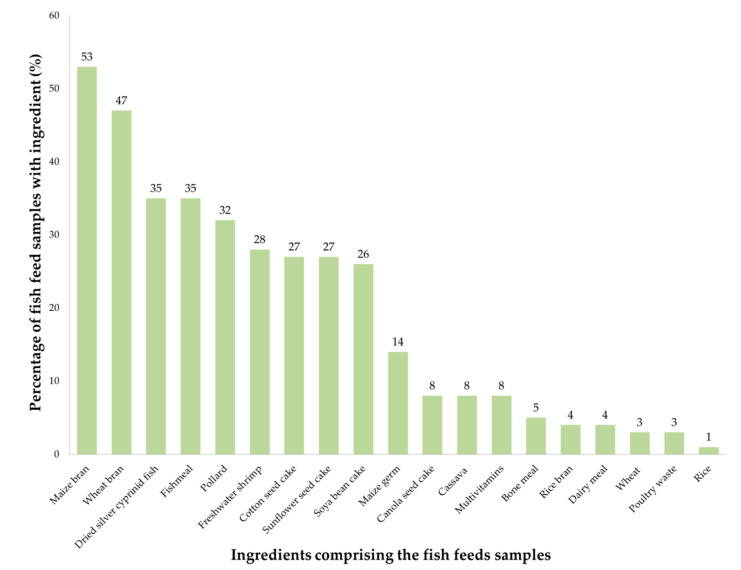
Percentage of fish feed samples with feed ingredients.

Different combinations of 19 feed ingredients comprised the fish feed samples collected and tested (Figure 1). Ingredients with the highest occurrence in the feed samples were maize bran (53%) and wheat bran (47%).

The 78 feed samples were categorized into 48 (62%) complete feeds, 19 (24%) complementary feeds and 11 (14%) ingredients. Eighteen (23%) samples were rainbow trout feeds or feed ingredients while 60 (77%) were for tilapia. Fifty-nine (76%) samples were obtained from fish farmers while 19 (24%) were obtained from fish feed manufacturers. Fifty-four (69%) samples were commercial feeds while 24 (31%) were homemade feeds prepared by fish farmers.

### 2.2. Mycotoxin Prevalence

Seventy-six (97%) fish feed samples tested positive for the presence of one or more mycotoxins. The prevalence of the mycotoxins tested ranged between 0–91% (Table 1). Mycotoxins with the highest prevalence were enniatin B (91%), deoxynivalenol (76%) and fumonisin B1 (54%).

More than half the mycotoxins (29/40 = 73%) tested for were detected in the samples. All samples tested negative for T-2 toxin (T2); T-2 triol; T-2 tetraol, 3-acetyldeoxynivalenol (3DON); 15-acetyldeoxynivalenol (15ADON); 2-amino-14,16-dimethyloctadecan-3-ol (AOD); diacetylscripenol (DAS); acetamido-butenolide (BUT); aflatoxin B2 (AFB2); aflatoxin G2 (AFG2) and ochratoxin (OTA).

**Figure 2:**
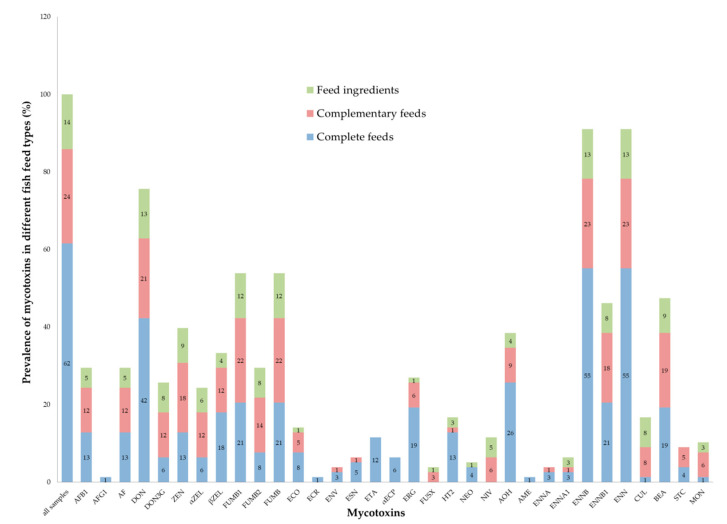
Prevalence of mycotoxins in different types of fish feed samples.

The prevalence of mycotoxins found in the different feed types is shown in Figure 2 and in Table S1.

**Figure 3:**
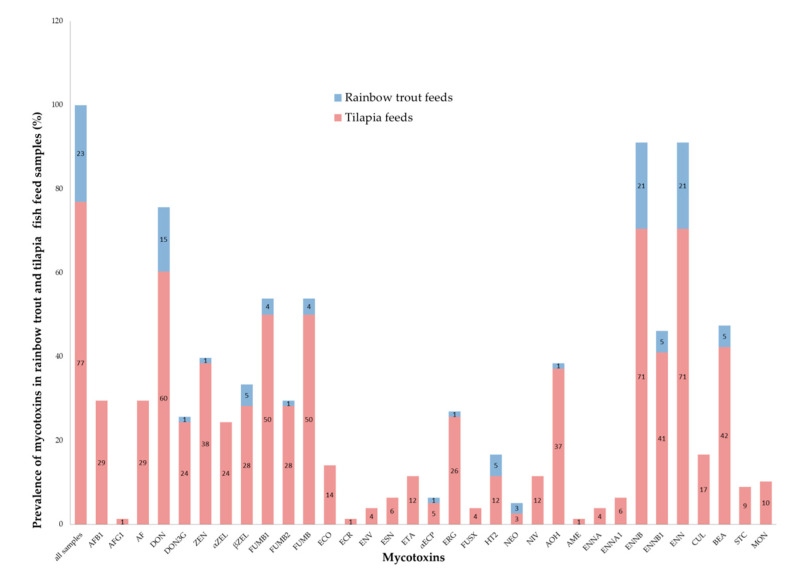
Prevalence of mycotoxins in rainbow trout and tilapia fish feed samples.

Figure 3 and Table S2 shows the prevalence of mycotoxins found in rainbow trout and tilapia fish feed samples.

**Figure 4:**
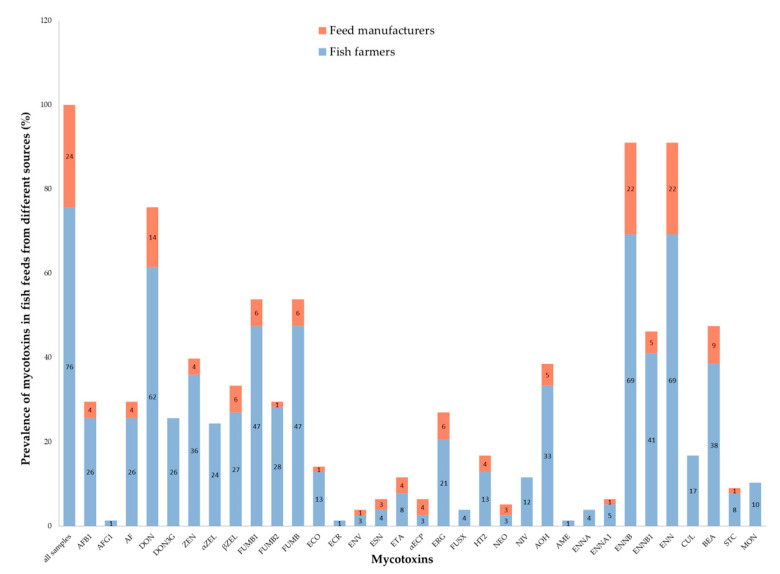
Prevalence of mycotoxins in fish feed samples from different sources.

The prevalence of mycotoxins found in fish feed samples collected from fish farmers and feed manufacturers is shown in Figure 4 and Table S3.

**Figure 5:**
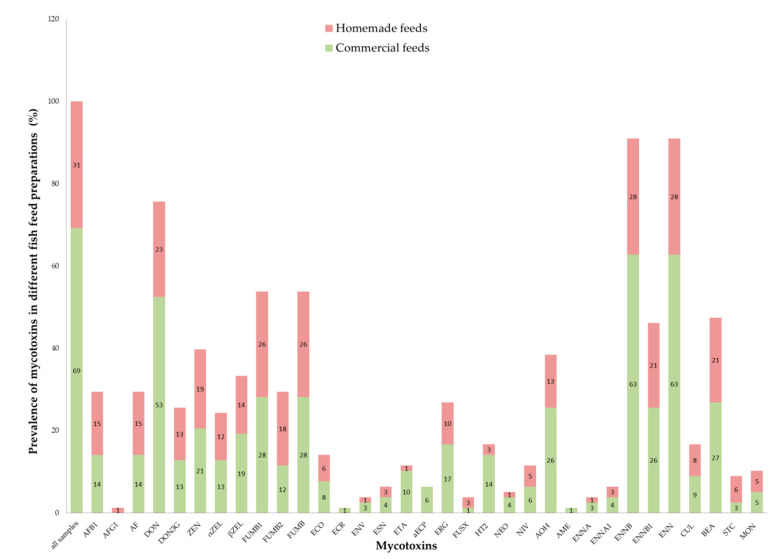
Prevalence of mycotoxins in different fish feed sample preparations.

Figure 5 and Table S4 shows the prevalence of mycotoxins found in commercial feeds and homemade feeds prepared by fish farmers.

### 2.3. Mycotoxin Levels

The levels of the mycotoxins tested are shown in Table 1. Mycotoxins with the highest level ranges were sterigmatocystin (STC) (<30.5–3517.1 µg/kg); moniliformin (MON) (<218.9–2583.4 µg/kg); ergotamine (ETA) (29.3–1895.6 µg/kg); fumonisin B1 (FUMB1) (<63.0–1427.4 µg/kg); beauvericin (BEA) (<15.9–841.8 µg/kg) and DON (<40.4–819.9 µg/kg). With regard to central tendency parameters, mycotoxins with the highest median levels were MON (530.4 µg/kg), CUL (141.6 µg/kg) and FUMB1 (116.8 µg/kg) while those with the highest mean levels were MON (839.3 ± 818.5 µg/kg), STC (591.3 ± 1298.0 µg/kg) and ETA (301.5 ± 602.5µg/kg).

The levels of mycotoxins in each of the feed types are shown in Supplementary Materials: Table S1. Kruskal–Wallis tests showed that DON (χ^2^ = 12.35; *p* = 0.002), FUMB (χ^2^ = 6.49; *p* = 0.039) and BEA (χ^2^ = 6.29; *p* = 0.043) levels were significantly different among complete, complementary and ingredients feed types. Complete feeds had significantly lower median levels of DON (<40.4 µg/kg), FUMB (<63.0 µg/kg) and BEA (<15.9 µg/kg) than complementary feeds (DON—z = −2.81, *p* = 0.002, median = 262.8 µg/kg; FUMB—z = −2.06, *p* = 0.020, median = 212.1 µg/kg and BEA—z = −2.32, *p* = 0.010, median = 42.5 µg/kg) and feed ingredients (DON—z = −2.80; *p* = 0.003, median = 208.2 µg/kg; FUMB—z = −2.24, *p* = 0.013, median = 248.8 µg/kg and BEA—z = −1.81, *p* = 0.035, median = 69.2 µg/kg). 

The levels of mycotoxins in rainbow trout and tilapia feeds are shown in Supplementary Materials: Table S2. Mann–Whitney tests showed significant differences in DON (z = −3.13; *p* = 0.002) and BEA (z = −2.23; *p* = 0.026) levels where rainbow trout feeds had significantly lower median levels of DON (<40.4 µg/kg) and BEA (<15.9 µg/kg) than tilapia feeds (DON—122.8 µg/kg; BEA—37.3 µg/kg).

The levels of mycotoxins in fish feeds from fish farmers and feed manufacturers are shown in Supplementary Materials: Table S3. Mann–Whitney tests showed significant differences in DON (z = 3.79; *p* < 0.0001) and BEA (z = 2.24; *p* = 0.025) levels where feeds from manufacturers had significantly lower levels of DON (<40.4 µg/kg) and BEA (<15.9 µg/kg) than feeds from fish farmers (DON—122.9 µg/kg; BEA—20.3 µg/kg).

Mycotoxin levels in commercial and homemade fish feeds are shown in Supplementary Materials: Table S4. Mann–Whitney tests showed significant differences in DON (z = −3.01; *p* < 0.003) with commercial feeds having significantly lower median levels (<40.4 µg/kg) than homemade feeds (305.7 µg/kg).

All 23 samples positive for AF were tilapia feeds. Of these, ten (44%) were complete feeds, nine (39%) were complementary feeds and four (36%) were ingredients. All ten (100%) of the complete feeds had AFB1 levels above the 5 µg/kg maximum level (ML) allowed in complete tilapia feeds in Kenya [37]. Similarly, three (30%) complete feeds had total AF levels above the 10 µg/kg maximum level (ML) allowed in complete tilapia feeds in Kenya [37] (Table 2). However, only three (30%) complete feeds had AFB1 levels above the ML allowed in complete and complementary animal feeds set by the European commission (EC) [38]. None of the four AFB1 positive feed ingredients had levels above 20 µg/kg which is the ML for AFB1 allowed in feed ingredients by EC [38] (Table 2). One sample had an HT2 level higher than the 250 μg/kg indicative level (IL) set by EC for sum of T2 and HT2 [39]. All other samples had DON, FUMB, OTA and ZEN levels below the guidance values (GV) set by EC for regulated mycotoxins as shown in Table 2.

**Figure 6:**
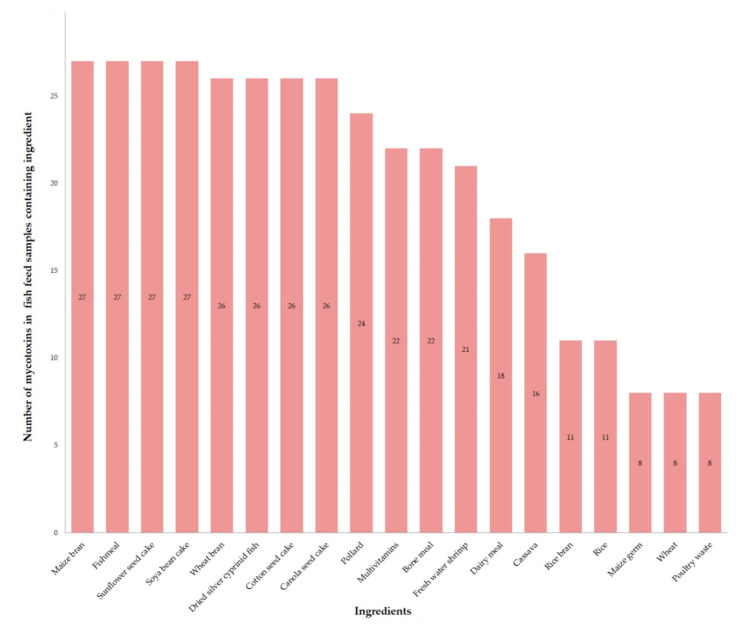
Number of mycotoxins in fish feed samples containing particular ingredients.

The prevalence and levels of mycotoxins in the fish feed samples categorized by feed ingredients are shown in Tables S5 and S6. Figure 6 shows the number of mycotoxins ranging from between 8 and 27 mycotoxins in the fish feed samples containing particular ingredients. Fish feed samples containing maize bran, soya bean meal, sunflower seed cake and fish meal comprised the highest number of mycotoxins.

Deoxynivalenol (DON) levels were significantly lower among feeds containing fish meal (z = 1.97, *p* = 0.05, median = <40.4 µg/kg), dried silver cyprinid (z = 2.34, *p* = 0.019, median = <40.4 µg/kg) and freshwater shrimp (z = 2.15, *p* = 0.032, median = <40.4 µg/kg) than those that did not contain these ingredients (fish meal median = 98.5 µg/kg; dried silver cyprinid median = 113.5 µg/kg; freshwater shrimp median = 83.1 µg/kg). 

### 2.4. Mycotoxins Co-Occurrence

**Figure 7:**
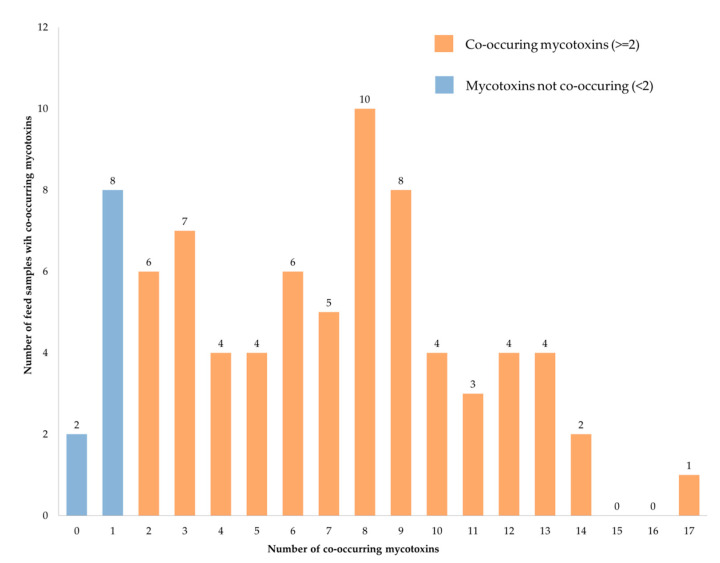
Co-occurrence of mycotoxins in fish feed and ingredient samples.

Sixty-eight (87%) feed samples tested positive for more than one mycotoxin (co-occurrence). The number of mycotoxins found in each sample ranged from 0 to 17. Ten (13%) of the samples had eight mycotoxins co-occurring in them while only one (1%) sample had as many as 17 mycotoxins co-occurring in it (Figure 7). The co-occurrence of eight mycotoxins was most common as observed in 10 (13%) of the samples.

Table 3 shows the prevalence of co-occurring regulated mycotoxins of economic importance detected in this study.

Correlation analysis was conducted to investigate how mycotoxin levels varied and if an association between them was observed. Spearman’s correlation test showed that some co-occurring mycotoxins were significantly associated. Correlations that were strong (*r_s_* ≥ 0.5) and occurred in more than 10 samples were considered noteworthy (Table 4).

### 2.5. Mycotoxin Dietary Exposure Estimates for Fish

Dietary exposure of mycotoxins by fish in the region was estimated based on the median levels of mycotoxins found in the feeds and calculating the probable daily intake (PDI) by the fish (Table 5). Sterigmatocystin (STC) had the highest concentration level (3517.1 µg/kg) of fish exposure but MON had the highest median (530.4 µg/kg). Similarly, based on a market fish size of 300 g [41,42] consuming 3.7 g per day [43], STC had the highest fish PDI of 43.4 µg/kg body weight per day (bw/day) while MON had the highest median (6.5 µg/kg bw/day). 

The median mycotoxin levels were below the no-observable adverse effects limit (NOAEL) for DON (600–800 µg/kg of feed) [44], ZEN (300 µg/kg of feed) [45] and HT2 (13 µg/kg bw/day) [46] and below the lowest-observed adverse effects limit (LOAEL) for FUMB (10,000 µg/kg of feed) [47] as set by European food safety authority (EFSA) for fish. However, two and five individual samples had levels above NOAEL for DON and ZEN respectively. 

There are no NOAELs and/or LOAELs set by EFSA for the other mycotoxins analysed due to the availability of scarce information on their toxicity [48,49,50,51,52,53,54] (Table 5).

## 3. Discussion

Seventy-eight fish feed samples were tested for 40 mycotoxins using HPLC-HRMS which, when employed together with a multi-toxin method, is a powerful technique for the concurrent determination of both modified and parent mycotoxins in feedstuffs. One advantage of the method used in this study, besides the ability to detect several mycotoxins simultaneously, was that it was not necessary to carry out a clean-up step after extraction. Additionally, it has been validated and has been used several times in previous studies [56,57,58]. However, there are drawbacks with multi-analyte methods that employ such an extract-and-shoot approach. Thus, problems with detection and quantification of an analyte can be encountered due to matrix effects and too low sample concentration are common [59,60]. In this study, high mycotoxin detection (LOD = 13–219 µg/kg) and quantitation (LOQ = 43–730 µg/kg) limits were experienced in some cases due to the complexity of the matrix of each sample tested. This effect potentially underestimated the prevalence and levels of some of the mycotoxins detected and quantified in the study. However, the results still reflect the occurrence of mycotoxins in fish feeds in Kenya and warrant the need to control mycotoxin contamination in fish feeds and fish exposure to mycotoxins in order to safeguard fish health.

Seventy-six samples (97%) tested positive for at least one mycotoxin. Streit et al. [61] and Greco, Pardo and Pose [62] obtained 100% prevalences in their studies of 139 mycotoxins in 83 feeds and feed ingredients from Europe and six mycotoxins in 28 rainbow trout feed from Argentina respectively.

A total of 29 of the 40 (73%) analysed mycotoxins were identified in 76 of the 78 samples positive for mycotoxin presence. The samples contained between 0 and 17 mycotoxins. The co-occurrence of eight mycotoxins was most common as observed in 10 (13%) of the samples. In a similar multi-mycotoxin study by Streit et al. [61], 43% (139) of 320 mycotoxins were detected in 83 feed samples collected in Europe. In this case, the co-occurrence of 28 mycotoxins was most commonly observed in nine (11%) of the samples. Co-occurrence of mycotoxins in feeds and feed ingredients is expected since they act as suitable mould substrates which may be invaded by more than one mould at a time thus producing multiple mycotoxins. Additionally, most of the moulds are capable of producing more than one mycotoxin, for example, *Fusarium culmorum* and *F. graminearum* may both produce DON and ZEN [9] and *F. poae* may produce ENN, BEA, BUT and CUL [63] in different environmental conditions.

Maize and wheat by-products, e.g., bran, pollard, maize germ and wheat grain, comprised most of the fish feed samples tested in this study. Worldwide, these ingredients are used to manufacture fish feeds [64,65]. These ingredients are prone to mycotoxin contamination such as DON [66], ZEN [67], FUM [67], OTA [68] and AF [69]. In this study, significantly lower levels of DON were identified in feeds containing fish byproducts, i.e., fish meal, dried silver cyprinid and freshwater shrimp than those without them. Fishmeal-based feeds usually contain less DON and ZEN compared to cereal-based feeds [65]. Fish byproducts have been used preferably as a source of protein for fish but their declining supply and high cost have led to efforts to find alternative protein sources from plant materials [70], e.g., soya bean, sunflower seeds, and cotton seeds, which are more prone to mycotoxin contamination [71]. 

Mycotoxin levels among three different types of feeds, that is, complete, complementary and ingredients were not significantly different except for DON, FUMB and BEA with the complete feeds having significantly lower levels than both complementary feeds and feed ingredients. Wheat and maize, whose by-products comprise most of the samples tested in this study (>47% and >53% respectively), are commonly contaminated with DON [66]. Similarly, fumonisins (FUM) are common contaminants of maize and maize byproducts [65]. It is therefore not surprising that higher levels of DON and FUMB were detected in complementary feeds and feed ingredients. Complete feeds comprise feed ingredients mixed together to obtain a composition containing all nutrients sufficient for a daily ration [72]. Some feed ingredients are more prone to mycotoxin contamination than others and if used for complete feed manufacture when contaminated with mycotoxins, there may be a dilution effect leading to lower levels in the complete feeds than in the original ingredients.

Fish species have varied sensitivities to different mycotoxins. Rainbow trouts are sensitive to AF [73] and DON [74] while catfishes tend to be resistant to AF [16] and DON [75]. Tilapia, on the other hand, are more sensitive to AF than catfishes [76] but more resistant to AF [16] and DON than rainbow trout [75]. In this study, rainbow trout feeds had significantly lower median levels of DON and BEA than tilapia feeds. This suggests that tilapia had a higher exposure risk to DON than rainbow trout in the study area. However, the levels observed in this study were not enough to cause adverse effects in tilapia as shown by Hooft et al. [75]. Although there is scarce information about sensitivity levels of fish to BEA, in vitro studies by García-Herranz et al. [77] showed cytotoxic effects in rainbow trout cells (RTH-149) indicating some level of sensitivity of trout to BEA.

Deoxynivalenol (DON) and BEA levels in samples from feed manufacturers were significantly lower than in those from fish farmers. Similarly, commercial feeds had significantly lower levels of DON than homemade feeds. The feed manufacturers produce the commercial feeds whereas the fish farmers purchase the commercial feeds for their fish or prepare homemade fish feeds from locally available ingredients. Factors that could possibly contribute to lower levels of mycotoxins in commercial feeds and feeds from manufacturers include: implementation of good manufacturing practices (GMP) [78,79]; shorter storage periods of raw ingredients and finished feeds and judicious selection of raw ingredients for feed production. On the other hand, fish farmers preparing homemade feeds may not have the skills or equipment to implement GMP and may also use raw ingredients that are locally available and whose quality and mycotoxin status are uncertain and varied. Additionally, those purchasing commercial feeds are often at the end of the feed supply chain where the feeds have undergone long storage [80] from the time they were manufactured or their raw ingredients harvested therefore having a longer risk period for mycotoxin contamination if storage conditions are poor.

The prevalence of total aflatoxins in this study was 29% with levels ranging from <14.7 to 93.6 µg/kg and a median and mean of <14.7 µg/kg and 14.2 ± 19.2 µg/kg respectively. Aflatoxin B1 had the highest prevalence while AFB2 and AFG2 were not detected. In an analysis of similar fish feeds from Nyeri county using an enzyme-linked immunosorbent assay (ELISA), a prevalence of 84% was obtained with levels ranging from 1.8 to 39.7 µg/kg and a median and mean of 3.6 µg/kg and 7.0 ± 8.3 µg/kg respectively [26]. The higher prevalence and lower means and medians in that study may be attributed to a lower limit of detection (LOD) of 1.75 µg/kg of the ELISA kit used [26] compared to the current study’s HPLC-HRMS LOD of 14.7 µg/kg. Similarly, Marijani et al. [33] reported higher mean levels (>90.1 µg/kg) and ranges (<2.0–806.9 µg/kg) in fish feeds from the Lake Victoria region in Kenya than in the current study possibly due to a lower LOD of 2 µg/kg. Other authors have reported varied prevalences, levels and ranges of various AFs in fish feed from different parts of the world [29,34,62,81,82,83]. In another study, Marijani et al. [34] have reviewed the toxic effects of AFs reported in assorted fish species ranging from reduced weight gain, reduced liver function to fish mortalities. Additionally, AFs have also been shown to accumulate in edible fish muscles [84] therefore indicating a risk of human exposure when consumed. Tumour formation has been reported in trout fed with feeds containing AF levels as low as 20 µg/kg for 4 weeks [85] therefore indicating that sub-acute to chronic fish exposure to AF levels as seen in this study may lead to carcinogenesis. Thirteen fish feed samples had AF levels above the ML allowed in fish feeds [37,38] indicating that these feeds are not safe for consumption by fish and may potentially lead to adverse health effects. 

In this study, DON was detected in 76% of the samples analysed with levels ranging from <40.4 to 819.9 µg/kg and a median and mean of 66.9 µg/kg and 168.9 ± 202.0 µg/kg respectively. However, Marijani et al. [33] reported a higher mean level of DON (201.4 µg/kg) but within a lower range (0–755.5 µg/kg) in fish feeds from the Lake Victoria region in Kenya while Rodrigues, Handl and Binder [29] observed a 48% prevalence of DON with a higher mean of 326 µg/kg and a higher median of 420 µg/kg in 25 feed commodities from Kenya. A few authors have reported the occurrence of DON in fish feeds from different parts of the world [62,65]. Deoxynivalenol (DON) causes acute gastroenteritis that leads to vomiting [86], feed refusal and bloody diarrhoea [87] in animals. Other signs of acute DON exposure include abdominal distress, increased salivation and malaise [88]. In fish, DON has been associated with decreased feed intake, low weight gains and growth rates in addition to liver lesions [74]. Although DON is less acutely toxic, its common occurrence in grains [89,90] and subsequently in animal feeds render it important enough to have regulations to control its presence in animal feedstuffs established [17,40]. Despite the mean and median DON levels obtained in this study being below the suggested European Food Safety Authority (EFSA) guidance value (GV) and NOAEL [44], 2 samples among the 78 tested had levels above NOAEL signifying a potential risk exposure and consequent adverse health effects to the fish consuming these feeds.

Deoxynivalenol (DON) exists in plants and feeds in several forms, including the acetylated precursors in the biosynthesis and the degraded or detoxified derivatives or metabolites formed by acetylation, glycosylation, de-epoxidation or oxidation to produce what is known as masked DON [91] A masked mycotoxin is a mycotoxin derivative undetectable by conventional analytical techniques because its structure has been changed in the plant [92]. Deoxynivalenol (DON) derivatives tend to occur together with DON but at much lower levels [93]. Among the commonly occurring masked DON derivatives are DON3G, 3ADON and 15ADON [92,94] which we tested for in this study.

Deoxynivalenol-3-glucoside (DON3G) was detected in 26% of the samples with levels ranging from <46.8 to 97.5 µg/kg and a median and mean of <46.8 µg/kg and 31.7 ± 20.9 µg/kg respectively. Streit et al. [61] reported a 75% prevalence of DON3G with a mean 15 µg/kg and a maximum of 7764 µg/kg in 83 feed samples collected in Europe. Being a DON derivative, DON3G is thought to cause similar effects as its parent mycotoxin, DON, such as emesis but to a lesser extent [95]. Deoxynivalenol-3-glucoside may be broken down to DON and a glucoside residue in the gastrointestinal tract of humans and animals therefore contributing to the overall exposure to DON [95,96].

In this study, acetylated derivatives of DON, 3ADON and 15ADON were not detected in any of the samples. Marijani et al. [33] reported combined 3ADON and 15ADON (ADONs) mean of 8.97 ± 4.65 µg/kg within a range of <2–63.2 µg/kg in fish feeds from Lake Victoria region in Kenya while Rodrigues, Handl and Binder [29] reported a 16% prevalence of ADONs with a mean of 67 µg/kg and median of 371 µg/kg in 25 feed commodities obtained from Kenya. Both 3ADON and 15ADON induce similar gastrointestinal lesions (necrosis of the crypt epithelial cells) and feed refusal and growth retardation as DON [97]. The acetylated DONs are metabolized to DON in the body giving the ADONs potentially the same risk as DON [97]. Pinton et al. [98] found that 15ADON is slightly more toxic and 3ADON is slightly less toxic than their parent DON.

The trichothecenes, FUSX, HT2, NEO and NIV, were detected at low prevalences (4%, 17%, 5% and 12% respectively) and median levels (<56.0, <41.6, <11.7 and 66.3 µg/kg respectively) in this study. Streit et al. [61] reported a higher prevalence of NIV (63%) but with a lower median level (17 µg/kg) in 83 samples from Europe. Nivalenol has been reported to decrease splenocyte numbers and cause lesions in the gastrointestinal tract, kidneys and spleen of pigs [48]. Mahdjoubi et al. [99] reported a higher prevalence of FUSX (45%) in maize and wheat from Algeria. Maize and wheat by-products especially bran, are common ingredients used to manufacture fish feeds in Kenya [26]. FUSX, an acetylated NIV [100], causes immunosuppression, intestinal malabsorption, developmental toxicity and genotoxicity [101]. Toxicity data on HT2 are very limited, however, since HT2 is a major metabolite of T-2 toxin, the toxicity of T2 in vivo is considered to include that of HT-2 toxin [102] and these include weight reduction, liver damage, reproductive toxicity, neurotoxicity as well as haematotoxic and immunotoxic effects [103]. In the current study, none of the samples had T2 or HT2 PDI levels higher than the NOAEL of 13 µg/kg bw/day [46] indicating that T2 and HT2 exposure levels may not be a current major concern in the area. Similarly, since NEO is also a metabolite of T2, it may present similar toxic effects to T2 although little evidence is provided in the literature [103]. Scarce information is available on the adverse health effects of these trichothecenes in fish. 

The prevalence of ZEN in this study was 40% with levels ranging from <38.0 to 757.9 µg/kg and a median and mean of 58.8 µg/kg and 136.0 ± 170.7 µg/kg respectively. Rodrigues, Handl and Binder [29] reported a higher prevalence of ZEN of 56% with a lower mean of 67 µg/kg and a higher median of 61 µg/kg in 25 feed commodities obtained from Kenya. Similarly, Greco, Pardo and Pose [33] reported a higher median of 88 µg/kg in rainbow trout feeds from Argentina. Zearalenone (ZEN) is normally found in the highest concentrations in maize [104] and the prevalence and concentrations may depend on the proportion of maize-derived feed samples in the study.

There was a strong and significantly positive correlation between ZEN and total ZELs (αZEL + βZEL; *r_s_* = 0.88, *n* = 22, *p* < 0.0001) and individual ZELs (αZEL-*r_s_* = 0.72, *n* = 17, *p* = 0.001 and βZEL-*r_s_* = 0.72, *n* = 16, *p* = 0.002) in this study. This indicates a direct relationship with a tendency for individual and total ZELs to increase with an increase in ZEN. A correlation occurs between the ZELs and ZEN since they are related whereby α and β ZEL are derivatives of ZEN mostly occurring in animal tissues [6,105]. However, αZEL and βZEL are also known to occur naturally in different plant substrates [106]. The ZELs are referred to as modified mycotoxins since they have had their structure modified by chemical or biological (plants, animals, microorganisms) processes while ZEN is a free mycotoxin which is the compound arising from the secondary metabolism of toxigenic fungi [6]. Zearalenone (ZEN) and its metabolites of reduction, αZEL and βZEL, have oestrogenic activity [105] causing reproductive disorders such as reduced embryonic survival, decreased foetal weight and decreased luteinizing hormone and progesterone production in animals [107]. Their oestrogenicity is ranked as follows: αZEL > ZEN > β ZEL with αZEL being more oestrogenic than ZEN while βZEL being less oestrogenic than ZEN [45]. In fish, ZEN is associated with oedema, estrogenic potency expressed as reproductive disorders and interferences in blood coagulation and iron-storage [107]. Similar to DON levels, the mean and median ZEN levels in this study were below GV and NOAEL suggested by EFSA [45]. However, five (16%) samples were above the 300 µg/kg NOAEL therefore raising a concern that exposed fish may suffer adverse health effects.

Total fumonisins B (FUMB) prevalence in this study was 54% with more FUMB1 (54%) being recorded than FUMB2 (29%). The FUMB levels ranged from <63.0 to 1427.4 µg/kg for FUMB1 and <68.9 to 649.2 µg/kg for FUMB2. The respective means and medians were 247.6 ± 331.9 µg/kg and 116.8 µg/kg for FUMB1 and 120.0 ± 158.4 µg/kg and <68.9 µg/kg for FUMB2. Rodrigues, Handl and Binder [29] reported a higher 76% prevalence of total FUMB with a higher mean of 956 µg/kg and a higher median of 670 µg/kg in 25 feed commodities obtained from Kenya. Similarly, Marijani et al. [33] reported higher FUMB1 and FUMB3 mean levels of 495.0 ± 201.0 µg/kg and 34.5 ± 13.9 µg/kg within a range of <2.0–2077 µg/kg and <2.0–137.2 µg/kg in fish feeds from Lake Victoria region in Kenya respectively. They did not detect any FUMB2 but detected FUMB3 unlike in our study.

Fumonisins B1 (FUMB1) and FUMB2 were positively and significantly correlated (*r_s_* = 0.60, *n* = 23, *p* = 0.003) showing a strong direct relationship where FUMB1 would increase as FUMB2 increases. Fumonisins are produced by *Fusarium* fungal species, some of which produce both FUMB1 and FUMB2, e.g., *Fusarium verticillioides* and *F. proliferatum* while others produce one or the other only, e.g., *F. subglutinans* produces FUMB1 only [108]. It appears that *Fusarium* species producing both FUMB1 and FUMB2 or those producing the individual fumonisins had access to the feed samples or their ingredients and produced both fumonisins. Fumonisins B1 usually occur more frequently (70–80%) than FUMB2 (15–25%) and FUMB3 (3–8%) [108]. This was similar to this study where FUMB1 had a higher prevalence than FUMB2. However, FUMB3 and other fumonisins were not measured in this study. Fumonisins (FUMs) cause liver and kidney tumours and are associated with leukoencephalomalacia in horses and swine lung oedema. In fish, FUM has been reported to cause reduced feed consumption and efficiency; vacuolation in nerve fibres and hepatocytes and lymphocytic infiltration of liver and brain parenchyma in catfish [109]. The levels observed in this study were well below the EU’s guidance values and NOAEL set by EFSA [47] suggesting that FUMBs might not yet be a concern in the study area, however, there is a need to continue monitoring their levels so that they do not exceed the set limits and therefore expose the fish to potential adverse health effects. 

The prevalence of total ergot alkaloids in the current study was 27% with levels ranging from <20.7 to 2055.3 µg/kg and a median and mean of 58.5 µg/kg and 175.5 ± 437.2 µg/kg respectively. Six ergot alkaloids were tested with ECO having the highest prevalence (14%) and ETA having the highest median (87.2 µg/kg) and mean (301.5 ± 602.5 µg/kg). In their study of 83 feed samples collected in Europe, Streit et al. [61] reported lower prevalences of ESN (4%) and ETA (4%) than in this study with higher median levels of ESN (27 µg/kg) and ECR (47 µg/kg) and a lower median of ETA (71 µg/kg). Ergot alkaloids have been reported to cause gangrene, central nervous system disorders (e.g., convulsions), abortions and death [110]. Matejova et al. reported circulatory failure in organs and tissues; dystrophy of gill lamellae and occurrence of cellular polymorphonuclear subepithelial infiltrates in the renal tubuli in carp fish orally exposed to 30% and 50% ergot alkaloids [111].

Total enniatins (ENN) prevalence in this study was 91% with more ENNB (91%) being recorded than ENNA (4%), ENNA1 (6%) and ENNB1 (46%). The ENN levels ranged from 19.4 to 186.7 µg/kg with ENNB recording the highest mean (41.9 ± 36.5 µg/kg) and ENNB1 recording the highest median (23.2 µg/kg) levels. Streit et al. [61] reported a higher prevalence of total ENN (96%) with a prevalence ranking of ENNA1 (95%) > ENNB and ENNB1 (92%) > ENNA (87%) > ENNB2 (10%) > ENNB3 (8%) and median ranking of ENNB1 (14 µg/kg) > ENNB (11 µg/kg) > ENNA1 (5.5 µg/kg) > ENNA and ENNB2 (0.8 µg/kg) > ENNB3 (0.01 µg/kg). Similarly, in this study, ENNB and ENNB1 had a higher prevalence than the other ENNs [49]. Enniatins are a common finding in foods, feeds and grains, however, their toxicity is generally low in vivo [112]. Conversely, in vitro activities of ENNB recorded include cytotoxicity, apoptosis, estrogenic activity, oxidative stress and genotoxicity [113]. As far as the authors know, there are no published in vivo toxicity data for the effects of enniatins in fish. The European food safety authority (EFSA) concluded that acute exposure to ENN was not a concern to human health, however, chronic exposure may be a concern [113]. This conclusion may also apply to animal health.

Enniatin B (ENNB) and ENNB1 were positively and significantly correlated (*r_s_* = 0.77, *n* = 36, *p* < 0.0001) showing a moderate direct relationship where ENNB would increase as ENNB1 increases. Enniatins are produced by *Fusarium* species of fungi. The infestation of feeds or feed ingredients with *Fusarium avenaceum* and/or *F. oxysporum* may explain the co-occurrence of ENNA, ENNA1, ENNB and ENNB1 since these fungi are capable of producing the four enniatins analysed [114].

In this study, AOH and its derivative AME had prevalences of 38% and 1% respectively with means of 18.1 ± 43.3 µg/kg and 94.5 µg/kg and medians of <36.2 µg/kg and 94.5 µg/kg respectively. Conversely, Streit et al. [61] reported a higher prevalence of 80% for AOH and 82% for AME. However, they reported lower median levels of 2.8 µg/kg for AOH and 1.4 µg/kg for AME. On the other hand, Marijani et al. [33,34] reported a very low AOH prevalence of 4% with a mean level of 91.3 ± 25.3 µg/kg in fish feeds from the East Africa region. Acute toxicity with AOH is low, however, genotoxic and oestrogenic effects have been reported in vitro [115]. In fish, Dellafiora et al. [116] reported low oestrogenic activity of AOH on trout in silico.

Beauvericin (BEA), MON, BUT, CUL and STC were reported in high prevalence and levels in this study. These compounds, including ENN, AOH and AME, are typically referred to as emerging mycotoxins since they are neither routinely determined [112], nor legislatively regulated [21,117]. Although the prevalence of MON and STC obtained in our study were low, the levels isolated were very high reaching up to 2583.4 µg/kg and 3517.1 µg/kg respectively. Streit et al. [61] reported a higher prevalence of BEA (98%), MON (76%) and BUT (52%) compared to this study. However, these high prevalences were paired with lower median levels of BEA (6.7 µg/kg) and MON (45 µg/kg) compared to this study. Neither BUT nor its analogues were detected in this study. Mahdjoubi et al. [99] also reported a higher prevalence of BEA (76.6%) in maize and wheat from Algeria.

Moniliformin (MON) has been reported to cause damage to the heart muscle, respiratory distress, decreased feed intake, decreased body weight gains and impaired immune function in animals [112]. No critical adverse health effects have been identified in fish exposed to MON via feed although a reduction in weight gain has been recorded in channel catfish [51]. In fish, 1.6 µg/kg body weight STC has been reported to cause a decrease in body weight and damage to erythrocytes and kidneys [118]. Sterigmatocystin (STC) is a precursor of AF [119] and its detection in this study suggest that the feeds may have been infested with *Aspergillus nidulans* or other *Aspergillus, Bipolaris* and *Chaetomium* species whose end metabolites are STC, and/or AF producing *Aspergillus* species such as *Aspergillus flavus*, *A. nomius* and *A. parasiticus* where the STC identified was an intermediate metabolite [119]. The mycotoxin with the highest level in this study was STC and being a precursor of AF, this signifies a potential problem with subsequent high levels of aflatoxins. 

Beauvericin (BEA) acts as an endocrine disruptor and has cytotoxic activity [120]. The European food safety authority (EFSA) concluded that acute exposure to BEA is not a concern to human health [112]. No such conclusion has been made about exposure in animals and less in fish although this may be applied to them. 15-hydroxy-culmorin (CUL) is a metabolite of culmorin which is said to have antifungal and phytotoxic activities but has low toxicity to animals cells [121,122]. This may also apply to fish although its co-occurrence with other mycotoxins may alter its toxicity. In this study, CUL was directly, strongly and positively correlated to total ENN (*r_s_* = 0.58, *n* = 13, *p* = 0.037) suggesting that there would be an increase in total ENN with an increase in CUL. It is possible that the correlation observed could be because some *Fusarium* species, for example, *F. poae* [123], *F. langsethiae* and *F. sporotrichioides* are capable of producing both ENN and CUL and its metabolites [124].

Mycotoxins are classified loosely as major and minor mycotoxins. Major mycotoxins are those that occur frequently in foods and feeds and are of health and economic importance. They include aflatoxins, ochratoxins, trichothecenes (deoxynivalenol and nivalenol), zearalenone, fumonisin [125] and their metabolites [126]. Although T-2 toxin and ergot alkaloids are not frequently isolated in foods and feeds, they are also classified as major because of their adverse effects on human and animal health. Many countries have set out regulatory measures to minimize the occurrence of major mycotoxins in foods and feeds. They have set maximum limits for mycotoxins in these products beyond which deem the foods and feeds undesirable for consumption by man and animals [127,128].

In this study, one, three and ten samples had mycotoxin levels above the indicative levels (IL) set for HT2 and maximum levels (ML) set for AF and AFB1 respectively. This signifies that fish consuming such feeds are exposed to potentially harmful levels of mycotoxins which may lead to adverse health effects due to mycotoxicosis. On the other hand, the levels of the other regulated mycotoxins, i.e., DON, OTA, ZEN and FUMB were below the GVs set by the European Union [72] and may not cause immediate mycotoxicoses on consumption. However, consumption of these low levels of mycotoxins over a long period of time may lead to chronic mycotoxicoses in the fish [16]. It is therefore important to avoid having these mycotoxins in feeds if possible or to keep the levels very low to avoid adverse health effects due to chronic mycotoxicoses.

Although minor mycotoxins are not frequently isolated in foods and feeds and are therefore not regulated, they may still pose a health risk to humans and animals that consume them. Minor mycotoxins of importance include: citrinin, cyclopiazonic acid, sterigmatocystin (STC), moniliformin (MON), gliotoxin, citreoviridin, tremorgenic mycotoxins, penicillic acid, roquefortine (ROQ), 3-nitropropionic acid, and fusaproliferin [126]. Other mycotoxins tested that may fall into this minor group of mycotoxins include: BUT; BEA; DAS; ENNA; ENNA1, ENNB; ENNB1; AOH; HT2; FUSX; NEO; CUL; AOD and their metabolites. Many of these less-studied mycotoxins, e.g., MON, BEA, ENN and BUT have been found to have generally low toxicities in vivo [112] and no conclusive evidence is present on their effects in animal health and even less in fish health [48,49,52,55,129]. 

The co-occurrence of multiple mycotoxins in compounded feeds and feed ingredients is not a strange finding. Detection of one mycotoxin in a sample leads to suspicion of the presence of one or more other mycotoxins [130]. Mycotoxin contamination of feeds and feed ingredients can occur when there is an infestation of the feed or ingredient with a fungus that produces multiple mycotoxins [131] or when a single ingredient is infested by multiple fungi which produce different mycotoxins or when separate ingredients containing one or more different mycotoxins are mixed together during feed manufacturing [130]. Additionally, the interaction between fungal species may influence mycotoxin production with some species stimulating while others inhibiting mycotoxin production.

The isolation of some *Fusarium* species in Kenyan wheat [19], for example, *F. poae* and *F. avenaceum* that produce ENN [132] or *F. equiseti* and *F. graminearum* that produce BUT in wheat, may account for the presence of these mycotoxins in our feed samples since the fungi responsible for their production are prevalent in Kenya. This maybe the first study on emerging mycotoxins in fish feeds in Kenya. However, emphasis have not been laid on these emerging mycotoxins since most have been reported to have low toxicity in vivo [112].

Meta-analysis of several studies by Smith et al. [20] found that the commonly co-occurring mycotoxins in Africa were: AF + OTA (35.7%), AF + FUM (28.6%); AF + ZEN (28.6%) and AF + OTA + ZEN (21.4%). In this study, OTA was not detected. This may partly be due to a higher LOD (27.3 µg/kg). However, the co-occurrence of AF + FUM at 24.4% and AF + ZEN at 15.4% were much lower than in Smith et al. study [20]. Gruber-Dorninger, Jenkins and Schatzmayr [18] reported co-occurrence of DON + ZEN at 69.1% with a positive correlation of 0.32 which was higher than the co-occurrence of 35% with a correlation of 0.46 (*p* < 0.0001) observed in the current study. Deoxynivalenol (DON) and ZEN are produced by the same fungi, *Fusarium culmorum* and *F. graminearum* [9] depending on the prevailing environmental factors therefore it is not strange to find co-occurrence in feeds and feed ingredients. Marijani et al. [33] found more samples (54.2%) with AF + FUMB co-occurrence than in this study (24.4%). They also reported DON + FUMB co-occurrence of 50%, similar to the 49% observed in this study.

Toxic effects, lesions and clinical signs observed in animals exposed to multiple mycotoxins are diverse, complex [130] and unpredictable even when based upon their individual toxicities [131]. Interaction of co-occurring mycotoxins within foods and feeds can predispose animals and man to additive, synergistic or even antagonistic toxic effects of the mycotoxins [20,131]. Data on the in vivo combined toxic effects of mycotoxins are limited [131] however, several in vitro studies have recorded various mycotoxin interactions in assorted animal cell lines [20,133,134,135,136,137,138,139,140,141,142]. Grenier and Oswald [131] have reviewed different in vivo studies reporting multi-mycotoxin interactions especially of AF and other mycotoxins in various species of animals. 

A previous study of aflatoxins in fish feeds and their effects on fish in Nyeri county [26], reported swollen abdomens, muscular haemorrhages, enlarged hearts, enlarged kidneys and enlarged livers with cystic swellings, necrosis and haemorrhages in rainbow trout. Farmers had also reported fish mortalities, poor appetites, poor growth rates and tumour-like lesions in their tilapia and rainbow trout. These were thought to be due to aflatoxin exposure but these findings could also have been due to exposure and/or co-exposure with other mycotoxins, which manifest similar adverse health effects. Mulei et al. [143], who were assessing the occurrence of infectious pancreatic necrosis (IPN) in a sub-group of the same population of tilapia and rainbow trout as in this study, reported the occurrence of clinical signs and lesions such as abnormal swimming, aggregation at the water inlet, vertebral scoliosis, pale gills, haemorrhages along the spinal column and haemorrhagic enteritis which were not attributed to IPN. These signs and lesions may be attributed to exposure and/or co-exposure to AFs and other mycotoxins. The infection with the IPN virus in this population might also suggest immunosuppression in the fish caused by mycotoxin exposure.

## 4. Conclusions

This study verified that mycotoxins in fish feeds and feed ingredients are prevalent (97%) and occur either singly (10%) or simultaneously (87%) with up to 17 toxins in one feed sample. Additionally, the detection of mycotoxin precursors, e.g., STC a precursor of AF, FUSX a precursor of NIV and mycotoxin metabolites, e.g., HT2 and NEO metabolites of T2, show the potential of co-occurrence which may further aggregate their toxic effects. Although the mean and median levels of the regulated mycotoxins, i.e., AF, DON, ZEN, FUMB and T2 + HT2 were lower than ML, GVs and IL, ten and one feed samples exceeded the ML and IL for AFB1 and HT2 respectively, therefore expressing their possible health effects in fish under chronic exposure. Additionally, the occurrence of these regulated mycotoxins together with minor, emerging or even masked mycotoxins may lead to additive, synergistic or antagonistic health effects in the fish. Furthermore, due to the immunosuppression effect of some of the mycotoxins detected, fish exposed to these mycotoxins may succumb to other disease conditions caused by bacterial, fungal or viral agents. Moreover, some of these mycotoxins accumulate in fish tissue and may pose a risk to humans and animals that consume them. It is therefore important to reduce the presence and levels of these mycotoxins in Kenya so as to minimize the risk of mycotoxicoses in fish and in other animals and human beings in the form of mycotoxin residues.

## 5. Materials and Methods

### 5.1. Samples

Using the snowball approach, fish farms and fish feed manufacturing plants located in Nyeri, Busia, Homa Bay, Migori, Kisumu, Vihiga and Siaya counties of Kenya were identified and 78 fish feeds and ingredients were randomly collected in the year 2015. Guided by Kenya’s KS ISO 6497:2002 standards on sampling of animal feeding stuffs [144], five incremental and representative feed samples were collected from each fish farm and manufacturer to obtain an aggregate sample of 1 kg total weight. The feed samples were packed in paper bags, wrapped in polythene bags and allocated unique identification numbers. The feed samples were transported to the laboratory and stored in a freezer at −20 °C until analysed at the Chemistry and Toxinology Research Group laboratories, Norwegian Veterinary Institute.

### 5.2. Reagents and Solutions

#### 5.2.1. Reagents

Acetonitrile (Romil Pure Chemistry Acetonitrile 190) (MeCN), deoxynivalenol (DON), 3-OAc-deoxynivalenol (3ADON), 15-OAc-deoxynivalenol (15ADON), deoxynivalenol-3-glucoside (DON3G), zearalenone (ZEN), α-zearalenol (αZEL), β-zearalenol (βZEL), ochratoxin A (OTA), T-2 toxin (T2), T-2 toxin triol, T-2 toxin tetraol, HT-2 toxin (HT2), nivalenol (NIV), neosolaniol (NEO), ergocristine (ECR), ergosine (ESN), α-ergocryptine (αECP), moniliformin (MON), fusarenon-X (FUS-X), diacetoxyscirpenol (DAS), alternariol (AOH), alternariol methyl ether (AME), sterigmatocystin (STC), beauvericin (BEA), 15-hydroxyculmorin (CUL), 5-acetamido-butenolide (BUT), 2-amino-14,16-dimethyloctadecan-3-ol (AOD), aflatoxin B1 (AFB1), aflatoxin B2 (AFB2), aflatoxin G1 (AFG1), aflatoxin G2 (AFG2), fumonisin B1 (FUMB1), fumonisin B2 (FUMB2) as well as the stable isotope-labelled analogues U-[^13^C-15]-NIV, U-[^13^C-15]-DON, U-[^13^C-21]-DON3G, U-[^13^C-17]-3ADON, U-[^13^C-17]-15ADON, U-[^13^C-22]-HT2, U-[^13^C-24]-T2, U-[^13^C-20]-OTA, U-[^13^C-18]-ZEN, U-[^13^C17]-AFB1, U-[^13^C17]-AFB2, U-[^13^C17]-AFG1 and U-[^13^C17]-AFG2 were provided by Romer labs (Tulln, Austria) [56,58]. Formic acid (>98%) (HCOOH), acetic acid (>99.8%) (CH_3_COOH) and ammonium acetate (>98%) (CH_3_COONH_4_) were provided by Merck KGaA (Darmstadt, Germany). LC-MS grade MeCN (Optima), LC-MS grade MeOH (Optima) and UHPLC-UV grade water (W6-212 water) were provided by Fisher Scientific (Loughborough, Leics., UK). The enniatins A (ENNA), A1 (ENNA1), B (ENNB), and B1 (ENNB1) and the ergot alkaloids, ergonovine (ENV), ergotamine (ETA), ergocornine (ECO), methysergide maleate (MSM) and bromocriptine mesylate (BCM) were provided as solids by Sigma-Aldrich (St. Louis, MO, USA) [56,58].

#### 5.2.2. Solutions Prepared

50% acetonitrile (50% MeCN): LC-MS grade acetonitrile (MeCN) and UHPLC-UV grade water (H_2_O) were mixed at a ratio of 50:50 *v*/*v*. This was prepared fresh prior to use.

Extraction solution (ES): Acetonitrile (MeCN), distilled and deionised water (H_2_O) and formic acid (HCOOH) were mixed at a ratio of 70:29.9:0.1 *v*/*v*/*v*. This was prepared fresh prior to extraction.

Stock solutions and intermediate solutions: Stock solutions of NIV, DON, 3ADON, HT2, T2, ZEN, DON3G, OTA, ECR, ESN, αECP and 15ADON were provided as solutions in acetonitrile (MeCN), ranging from 10 to 100 mg/L. Stock solutions of DON3G and 15ADON were further diluted in MeCN to obtain intermediate standard solutions of 10 mg/L. Stock solutions of the ergot alkaloids, ENV, ETA, ECO, MSM and BCM were prepared by dissolving the respective solids in MeCN or MeOH to concentrations of 100–500 mg/L. Similarly, stock solutions of the enniatins, ENNA, ENNA1, ENNB and ENNB1 were prepared by dissolving the respective solids in MeOH to 200 mg/L [58]. This was prepared fresh prior to extraction.

Spike standards (SS): Two sets of standard solution mixtures, i.e., set A containing DON, DON3G, 3ADON, 15ADON, ZEN, αZEL, βZEL, OTA, ECO, ECR, ENV, ESN, ETA, αECP, FUSX, T2, T2 triol, T2 tetraol, HT2, NEO, NIV, AOH, AME, AOD, ENNA, ENNA1, ENNB, ENNB1, CUL, DAS, BEA, BUT, STC and MON and set B containing AFB1, AFB2, AFG1, AFG2, FUMB1 and FUMB2, were prepared by mixing together the respective stock or intermediate standard solutions. The two sets were combined, the solvent evaporated and then re-dissolved in 50% MeCN to obtain a final concentration of 200 µg/L of each mycotoxin [56,58]. This was stored at −20 °C and was adjusted to room temperature (RT) and mixed thoroughly prior to use.

Internal standards (IS): This combined internal standard solution of 11 mycotoxins containing stable isotope-labelled analogues as well as semi-synthetic ergot alkaloids was prepared in MeCN/water (50:50, *v*/*v*) to produce a final concentration of 251 µg/L of U-[^13^C-18]-ZEN, 500 µg/L of U-[^13^C-22]-HT2, 443 µg/L of U-[^13^C-22]-T2, 506 µg/L of U-[^13^C-15]-DON, 502 µg/L of U-[^13^C-17]-3ADON, 500 µg/L of U-[^13^C-17]-15ADON, 500 µg/L of U-[^13^C-20]-OTA, 530 µg/L of U-[^13^C-15]-NIV, 530 µg/L of U-[^13^C-21]-DON3G, 500 µg/L of methysergide maleate and 624 µg/L of bromocriptine mesylate [58]. This was stored at −20 °C and was adjusted to room temperature (RT) and mixed thoroughly prior to use.

Mobile phase A (MPA): 5 mM ammonium acetate (CH_3_COONH_4_) was mixed with LC-MS grade water containing 0.1% of acetic acid (CH_3_COOH). This was prepared fresh prior to extraction.

Mobile phase B (MPB): 5 mM CH_3_COONH_4_ was diluted in 25 mL LC-MS grade water then mixed with 95% LC-MS grade MeOH containing 0.1% CH_3_COOH. This was prepared fresh prior to extraction.

### 5.3. Sample Preparation

The collected fish feeds and ingredients were brought to room temperature and mixed thoroughly. A subsample of 250 g of each was apportioned, mixed thoroughly and ground to a fine powder for 1 min at 7500 rotations per minute (rpm) using a grinder (Retsch Grandomix GM200, Haan, Germany). Using a weighing balance (Sartorious portable PT 1200, Goettingen, Germany), 2.5 g was weighed into a Falcon tube (Thermo Fisher Scientific, Waltham, MA, USA). Twenty millilitres (20 mL) of extraction solution was poured into the tube and mixed with the feed. The mixture was vortexed (VWR International, Milan, Italy) for 30 s and then shaken at 175 rpm for 30 min on an orbital shaker (Edmund Buhler Swip SM25, Hechingen, Germany). The mixture was then centrifuged (Beckman Coulter Allegra x30R, Brea, CT, USA) for 10 min at 4000 rpm at 4 °C. The supernatant was decanted into a fresh Falcon tube and refrigerated at 4 °C overnight.

Five hundred microliters (500 µL) of the supernatant were transferred into a microfilter tube (Costar Spin-X centrifuge filter tube 0.22 µm, Corning, Inc., Corning, NY, USA) and centrifuged (Thermo Scientific Micro 21R, Waltham, MA, USA) for 1 min at 4000 rpm at 4 °C. Forty microliters (40 µL) of the filtrate were transferred into chromatography vials (Thermo Fisher Scientific, Waltham, MA, USA) and mixed with the 10 µL of combined IS solution before HPLC-HRMS analysis. 

Two additional samples were prepared by weighing 2.5 g of mycotoxins-free fish feed into two Falcon tubes. One sample was extracted as described above and was used as a blank sample whereas the other sample was used to prepare a spiked sample by adding 50 µL of the combined spike standard solution (SS, 10 µg/mL, 50% MeCN) to the fish feed sample. It was allowed to stand for 15 min at room temperature under a laminar hood to evaporate the solvent before extraction was performed as described above. 

### 5.4. Analysis

Multi-mycotoxin analysis was performed using a Q-Exactive Hybrid Quadrupole-Orbitrap mass spectrometer including a heated electrospray ion source (HESI-II) and coupled to a Vanquish UHPLC system (Thermo Scientific, Waltham, MA, USA) as carried out by Ivanova et al. [56]. Chromatographic separation was achieved at 30 °C on a 150 × 2.1 mm Kinetex reversed-phase F5 column (2.6 µm, 100 Å; Phenomenex, Torrance, CA, USA). The flow rate of the mobile phases A and B was 0.25 µL/min, the injection volume was 1 µL and the total run time was 43 min. Gradient elution was employed starting at 5% MPB for 1 min, linearly increasing to 15% MPB in 15 min, to 79% MPB in 10 min, and finally, to 100% MPB in 10 min. After washing the column for 2 min with 100% MPB, the mobile phase was returned to the initial conditions and the column was eluted isocratically for 2.5 min [56]. Retention time and high-resolution mass detection were used to detect different mycotoxins. Quantification was completed by measuring the peak areas.

### 5.5. Method Evaluation and Quality Control

In order to assess method accuracy (based on analyte recovery), calculate limits of detection (LOD) and quantification (LOQ), and determine the signal suppression or enhancement (SSE) of individual toxins, a spiked control fish feed sample was extracted and analysed on three separate days.

The standard deviation (SD) of the y-intercept of the respective matrix-matched calibration curves was used to establish the LOD and LOQ of each of the 40 mycotoxins based on their corresponding slope(s) using the equations LOD = 3 × SD/s and LOQ = 10 × SD/s, respectively [145]. Signal suppression or enhancement, expressed as a percentage (SSE%), was calculated as a ratio of the slope of the matrix-assisted standard calibration curve to the calibration curve in MeCN/water (50:50). Signal suppression or enhancement values above 100% were considered as signal enhancement by the matrix whereas those below 100% were from signal suppression by the matrix. Recovery rates (R) were calculated for all mycotoxins as a percentage: 100 × (Mycotoxin concentration of spiked sample − Mycotoxin concentration of unspiked sample)/Concentration of the mycotoxin spiked.

The LODs for the 40 mycotoxins ranged from 13–219 µg/kg, whereas the LOQs ranged from 43–730 µg/kg based on matrix-matched calibration curves. Regression coefficients (*r*^2^) ranged from 0.989–1.000. The recovery rates ranged from 44–145%, while the SSE% ranged from 36–285% indicating in part substantial matrix effects during ionisation of the analytes (Table S7).

### 5.6. Data Analysis

Data processing including calculation of mycotoxin concentrations was performed using Xcalibur™ software (Version 2.2, Thermo Scientific, Waltham, MA, USA, 2011). Mycotoxin levels generated by Xcalibur were exported to Microsoft^®^ Excel 2007 (Microsoft Corporation, Redmond, WA, USA) and prepared for statistical analyses in Stata/SE 14.2 (StataCorp LLC, College station, TX, USA, 2016). 

Summary statistics were generated and numeric variables were expressed as range, percentiles, median and mean ± standard deviation. Mycotoxin levels below LOD were assigned half their respective LOD values (LOD/2) for the statistical calculations. The data were skewed to the right (lower concentrations) and therefore non-parametric tests such as Mann–Whitney, Kruskal–Wallis and Dunn’s tests were used to compare medians. Correlations between mycotoxins were established using Spearman’s rank correlation test. Observations were considered significant at *p* ≤ 0.05. 

Fish dietary mycotoxin exposure estimates to the analysed mycotoxins were evaluated by calculating the minimum, median and maximum probable daily intake (PDI) using the formula: PDI (µg/kg per bw/day) = [MC × FC]/BW where MC is the mycotoxin concentration (µg/kg); FC is the average feed consumption of a fish per day (g/day) and BW is the average fish table body weight (kg) [99,146]. In this study, an FC of 3.7 g/day [43] and a BW of 300 g market body weight [41,42] were used.

Mycotoxin exposure risk characterization for fish was carried out by comparing the mycotoxin levels found in the fish feeds and/or the estimated PDI with no-observed adverse effects limits (NOAEL) and/or lowest-observed adverse effects limit (LOAEL).

## Figures and Tables

**Table 1:** Mycotoxins prevalence and levels in fish feed and ingredient samples (*n* = 78). Mean calculated using limit of detection (LOD)/2 in samples where mycotoxins levels detected were less than LOD (x = number of positive samples).

Mycotoxins	Prevalence % (x)	Range µg/kg	Mean ± S µg/kg	10th Percentile µg/kg	25th Percentile µg/kg	Median µg/kg	75th Percentile µg/kg	90th Percentile µg/kg
Aflatoxins								
AFB1	29 (23)	<14.7–43.6	10.8 ± 8.4	<14.7	<14.7	<14.7	<14.7	17.2
AFG1	1 (1)	<155.8	<155.8	<155.8	<155.8	<155.8	<155.8	<155.8
AF	29 (23)	<14.7–93.6	14.2 ± 19.2	<14.7	<14.7	<14.7	<14.7	21.7
Deoxynivalenol and its metabolites								
DON	76 (59)	<40.4–819.9	168.9 ± 202.0	<40.4	<40.4	66.9	263.2	456.3
DON3G	26 (20)	<46.8–97.5	31.7 ± 20.9	<46.8	<46.8	<46.8	<46.8	66.0
Zearalenone and its metabolites								
ZEN	40 (31)	<38.0–757.9	136.0 ± 170.7	<38.0	<38.0	58.8	191.1	367.8
αZEL	24 (19)	<22.2–288.4	61.6 ± 76.1	<22.2	<22.2	26.7	79.4	161.6
βZEL	33 (26)	<16.0–79.8	31.3 ± 23.5	<16.0	<16.0	28.4	48.2	64.7
Fumonisins								
FUMB1	54 (42)	<63.0–1427.4	247.6 ± 331.9	<63.0	<63.0	116.8	302	622
FUMB2	29 (23)	<68.9–649.2	120.0 ± 158.4	<68.9	<68.9	<68.9	146.7	230.6
FUMB	54 (42)	<63.0–2076.6	313.3 ± 455.0	<63.0	<63.0	160.7	336.5	785.7
Ergot alkaloids								
ECO	14 (11)	37.6–64.3	47.5 ± 9.8	38.8	39.4	42.3	55.3	59.5
ECR	1 (1)	<24.9	<24.9	<24.9	<24.9	<24.9	<24.9	<24.9
ENV	4 (3)	<21.9	<21.9	<21.9	<21.9	<21.9	<21.9	<21.9
ESN	6 (5)	<38.4–144.2	48.1 ± 54.4	<38.4	<38.4	<38.4	38.5	101.9
ETA	12 (9)	<29.3–1895.6	301.5 ± 602.5	28.9	58.5	87.2	166.6	585.1
αECP	6 (5)	<41.0–81.3	32.7 ± 27.2	<41.0	<41.0	<41.0	<41.0	57.0
ERG	27 (21)	<20.7–2055.3	175.5 ± 437.2	<20.7	38.9	58.5	111.7	206.3
Other trichothecenes							
FUSX	4 (3)	<56.0	<56.0	<56.0	<56.0	<56.0	<56.0	<56.0
HT2	17 (13)	<41.6–411.8	60.7 ± 108.7	<41.6	<41.6	<41.6	<41.6	101.3
NEO	5 (4)	<177.7	<177.7	<177.7	<177.7	<177.7	<177.7	<177.7
NIV	12 (9)	<40.3–76.0	53.0 ± 24.8	<40.3	<40.3	66.3	69.8	72.2
Alternariol and its metabolites								
AOH	38 (30)	<36.2–43.3	18.9 ± 4.6	<36.2	<36.2	<36.2	<36.2	<36.2
AME	1 (1)	94.5	94.5	94.5	94.5	94.5	94.5	94.5
Enniatins								
ENNA	4 (3)	<26.1	<26.1	<26.1	<26.1	<26.1	<26.1	<26.1
ENNA1	6 (5)	<13.5–23.8	11.8 ± 7.6	<13.5	<13.5	<13.5	14.7	20.2
ENNB	91 (71)	<38.8–150.0	41.9 ± 36.5	<38.8	<38.8	<38.8	55.3	118.4
ENNB1	46 (36)	<12.9–43.5	23.0 ± 8.5	14.8	16.6	23.2	27	35.3
ENN	91 (71)	19.4–186.7	54.9 ± 44.2	19.4	19.4	36.8	73.3	121.9
Other mycotoxins							
CUL	17 (13)	<42.3–288.7	136.9 ± 73.5	59.9	84.1	141.6	185.4	216.1
BEA	47 (37)	<15.9–841.8	84.4 ± 148.3	<15.9	<15.9	34.4	87.9	216.7
STC	9 (7)	<30.5–3517.1	591.3 ± 1298.0	<30.5	<30.5	<30.5	280.4	1645.8
MON	10 (8)	<218.9–2583.4	839.3 ± 818.5	213.2	286.6	530.4	1192.3	1633.2

Note: For statistical analysis, levels below LOD were substituted with LOD/2 while actual values were used for levels between LOD and LOQ. **Key:** LOD, Limit of detection; LOQ, Limit of quantification; <, less than; SSE, Signal Suppression or Enhancement; µg/kg, micrograms per kilogram; %, per cent; *n* = total number of samples tested; x, number of positive samples; AFB1, aflatoxin B1; AFG1, aflatoxin G1; AF, total aflatoxins; DON, deoxynivalenol; DON3G, deoxynivalenol-3-glucoside; ZEN, zearalenone; αZEL, alpha zearalenol; βZEL, beta zearalenol; FUMB1, fumonisin B1; FUMB2, fumonisin B2; FUMB, total fumonisins B; ECO, ergocornine; ECR, ergocristine; ENV, ergonovine; ESN, ergosine; ETA, ergotamine; αECP, alpha ergocryptine; ERG, total ergot alkaloids; FUSX, fusarenon X; HT2, HT-2 toxin; NEO, neosolaniol; NIV, nivalenol; AOH, alternariol; AME, alternariol methyl ether; ENNA, enniatin A; ENNA1, enniatin A1; ENNB, enniatin B; ENNB1, enniatin B1; ENN, total enniatins; CUL, 15 hydroxy-culmorin; BEA, beauvericin; STC, sterigmatocystin; MON, moniliformin.

**Table 2:** Proportion of samples with contamination levels above maximum limits (ML), guidance values (GV) or indicative levels (IL) for mycotoxins in animal feedstuffs.

Regulated Mycotoxin	Feed Characteristic	ML/IL/GV	Level/Value (µg/kg)	Samples > ML/IL/GV % (x)	Ref.
AFB1	Complete tilapia feed	ML	5	43 (10) ^†^	[37]
	Animal feed materials	ML	20	0	[38]
	Complementary and complete animal feed	ML	10	13 (3) ^‡^	[38]
AF	Complete tilapia feed	ML	10	13 (3) ^‡^	[37]
OTA	Animal feed materials—cereals and cereal products	GV	250	0	[40]
DON	Animal feed materials—Cereals and cereal products and maize by-products	GV	8000–12,000	0	[40]
	Complementary and complete animal feedstuffs	GV	5000	0	[40]
Total FUMB	Animal feed materials—maize and maize products	GV	60,000	0	[40]
	Complementary and complete feedstuffs for fish	GV	10,000	0	[40]
ZEN	Animal feed materials—Cereals and cereal products and maize by-products	GV	2000–3000	0	[40]
T2 + HT2	Cereal products for animal feed	IL	500–2000	0	[39]
	Compound animal feed	IL	250	1 (1) ^π^	

^†^ All 10 complete feeds were above 5 µg/kg; ^‡^ 3 of the 67 compound feeds were above 10 µg/kg. ^π^ 1 of the 67 compound feeds was above 250 µg/kg.

**Table 3:** Prevalence of co-occurring regulated mycotoxins in fish feed samples (*n* = 78).

Regulated Mycotoxins	AFB1 % (x)	AFG1 % (x)	DON % (x)	ZEN % (x)	FUMB1 % (x)	FUMB2 % (x)	HT2 % (x)	Total AF % (x)	Total FUMB % (x)
AFB1	30 (23)								
AFG1	<1 (1)	<1 (1)							
DON	28 (22)	<1 (1)	76 (59)						
ZEN	15 (12)	<1 (1)	35 (27)	40 (31)					
FUMB1	24 (19)	<1 (1)	49 (38)	36 (28)	54 (42)				
FUMB2	13 (10)	<1 (1)	26 (20)	26 (20)	30 (23)	30 (23)			
HT2	6 (5)	<1 (1)	17 (13)	5 (4)	9.0 (7)	-	17 (13)		
Total AF	30 (23)	<1 (1)	28 (22)	15 (12)	24 (19)	5 (4)	6 (5)	30 (23)	
Total FUMB	24 (19)	<1 (1)	49 (38)	40 (28)	54 (42)	10 (8)	9 (7)	24 (19)	54 (42)

**Table 4:** Correlation of mycotoxins within fish feed samples (*n* = 78).

Mycotoxin 1	Mycotoxin 2	Prevalence	Correlation Coefficient	
		% (x)	*r_s_*	*p*-Value
ENNB1	ENNB	46 (36)	0.77	<0.0001
FUMB1	FUMB2	30 (23)	0.60	0.0026
Total ZEL	ZEN	28 (22)	0.88	<0.0001
ZEN	αZEL	22 (17)	0.72	0.0012
ZEN	βZEL	21 (16)	0.72	0.0016
ENN	CUL	17 (13)	0.58	0.0368

Key: x, number of samples positive for both mycotoxins; *r_s_*, Spearman’s correlation coefficient.

**Table 5:** Estimated mycotoxin daily intake for a market fish size of 300 g consuming 3.7 g of feed per day against no-observable adverse effects limit (NOAEL) and/or lowest-observed adverse effects limit (LOAEL) set by the European food safety authority (EFSA).

Mycotoxin	Levels in Feed (µg/kg)		NOAEL	LOAEL	PDI (µg/kg bw/day)		NOAEL	LOAEL	Toxic Endpoint Reported by EFSA	Ref.
	Range	Median	(µg/kg Feed)		Range	Median	(µg/kg bw/day)			
AFB1	<14.7–43.6	<14.7			<0.18–0.54	<0.18				
AFG1	<155.8	<155.8			<1.92	<1.92				
AF	<14.7–93.6	<14.7			<0.18–1.15	<0.18				
DON	<40.4–819.9	66.9	600–800 ^‡^		<0.50–10.11	0.83			Decreased feed intake, body weight gain, growth rate, feed and efficiency, retained nitrogen, recovered energy, energy retention efficiency and nitrogen retention	[44]
DON3G	<46.8–97.5	<46.8	n.s.	n.s.	<0.58–1.20	<0.58	n.s.	n.s.		[44]
ZEN	<38.0–757.9	58.8	300^β^		<0.47–9.35	0.73	9 ^β^		Decreased number of monocytes, increased number of granulocytes and increased lipid peroxidation in liver and gill and altered the carbohydrate metabolism.	[45]
αZEL	<22.2–288.4	26.7	n.s.	n.s.	<0.27–3.56	0.33	n.s.	n.s.		[45]
βZEL	<16.0–79.8	28.4	n.s.	n.s.	<0.20–0.98	0.35	n.s.	n.s.		[45]
FUMB1	<63.0–1427.4	116.8	10,000 ^α^–20,000 ^β^	10,000^π^	<0.78–17.6	1.44	400 ^α^	500 ^π^	Reduced weight gain in Nile tilapia Pathological alterations in liver, pancreas, kidney, heart and brain, changes in haematological parameters and reduced body weight gain in carp Reduced body weight gain and microscopic liver lesions in catfish	[47]
FUMB2	<68.9–649.2	<68.9			<0.85–8.01	<0.85				
FUMB	<63.0–2076.6	160.7	10,000^α^–20,000^β^	10,000^π^	<0.78–25.61	1.98	400 ^α^	500 ^π^	Reduced weight gain in Nile tilapia Pathological alterations in liver, pancreas, kidney, heart and brain, changes in haematological parameters and reduced body weight gain in carp Reduced body weight gain and microscopic liver lesions in catfish	[47]
ECO	37.6–64.3	42.3			0.46–0.79	0.52				
ECR	<24.9	<24.9			<0.31	<0.31				
ENV	<21.9	<21.9			<0.27	<0.27				
ESN	<38.4–144.2	<38.4			<0.47–1.78	<0.47				
ETA	<29.3–1895.6	87.2			<0.36–23.38	1.08				
αECP	<41.0–81.3	<41.0			<0.51–1.00	<0.51				
ERG	<20.7–2055.3	58.5			<0.26–25.35	<0.72				
FUSX	<56.0	<56.0			<0.69	<0.69				
HT2	<41.6–411.8	<41.6			<0.51–5.08	<0.51	13 ^†,π^		Reduced feed intake, growth and haematocrit values as well as increased mortality	[46,55]
NEO	<177.7	<177.7			<2.19	<2.19				
NIV	<40.3–76.0	66.3	n.s.	n.s.	<0.50–0.94	0.82	n.s.	n.s.		[48,55]
AOH	<36.2–43.3	<36.2			<0.45–0.53	<0.45				
AME	94.5	94.5			1.17	1.17				
ENNA	<26.1	<26.1	n.s.	n.s.	<0.32	<0.32	n.s.	n.s.		[49]
ENNA1	<13.5–23.8	<13.5	n.s.	n.s.	<0.17–0.29	<0.17	n.s.	n.s.		[49]
ENNB	<38.8–150.0	<38.8	n.s.	n.s.	<0.48–1.85	<0.48	n.s.	n.s.		[49]
ENNB1	<12.9–43.5	23.2	n.s.	n.s.	<0.16–0.54	0.29	n.s.	n.s.		[49]
ENN	19.4–186.7	36.8	n.s.	n.s.	0.24–2.30	0.45	n.s.	n.s.		[49]
CUL	<42.3–288.7	141.6			<0.52–3.56	1.75				
BEA	<15.9–841.8	34.4	n.s.	n.s.	<0.20–10.38	0.42	n.s.	n.s.		[49]
STC	<30.5–3517.1	<30.5			<0.38–43.38	<0.38				
MON	<218.9–2583.4	530.4	n.s.	n.s.	<2.70–31.86	6.54	n.s.	n.s.		[51]

^‡^ based on carp and rainbow trout; ^†^ total of T2 and HT1; ^α^ based on Nile tilapia; ^β^ based on carp; ^π^ based on catfish; n.s.—not set. Key: NOAEL, no observable adverse effects limit; LOAEL, lowest observable adverse effects limit; PDI, probable dietary intake; Ref., references; µg/kg, micrograms per kilogram; µg/kg bw/day, micrograms per kilogram body weight per day.

## Supplementary Materials

The following are available online at https://www.mdpi.com/2072-6651/12/10/627/s1, Table S1. Mycotoxins prevalence and levels in complete feeds, complementary feeds and feed ingredients samples; Table S2. Mycotoxins prevalence and levels in rainbow trout and tilapia feeds samples; Table S3. Mycotoxins prevalence and levels in fish feeds samples from fish farmers and feed manufacturers; Table S4. Mycotoxins prevalence and levels in commercial and homemade fish feeds samples; Table S5. Prevalence of mycotoxins in fish feed samples containing particular ingredients; Table S6. Median mycotoxin levels in fish feed samples containing particular ingredients; Table S7. Performance parameters of the multi-mycotoxin HPLC-HRMS method used.
