# Supplementary material for: Co-Occurrence and Levels of Mycotoxins in Fish Feeds in Kenya
Source: Toxins (Basel). 2020 Sep 30;12(10):627. doi: 10.3390/toxins12100627 (PMC7600487; doi:10.3390/toxins12100627)
Supplement: Supplementary file 1 [file toxins-12-00627-s001.pdf]

Supplementary Materials: Co-Occurrence and Levels of Mycotoxins in Fish Feeds in Kenya

Evalyn Wanjiru Mwihia, Jan Ludvig Lyche, Paul Gichohi Mbuthia, Lada Ivanova, Silvio Uhlig, James K. Gathumbi, Joyce G. Maina, Eric Emali Eshitera and Gunnar Sundstøl Eriksen

Table S1. Mycotoxins prevalence and levels in complete feeds, complementary feeds and feed ingredient samples.

Mycotoxin	Feed Type	Prevalence % (x)	Range µg/kg	Mean ± SD µg/kg	10th Percentile µg/kg	25th Percentile µg/kg	Median µg/kg	75th Percentile µg/kg	90th Percentile µg/kg
**AFB1**	Complete	43 (10)	<14.7–43.6	13.4 ± 11.8	<14.7	<14.7	<14.7	13.6	24.9
	Complementary	39 (9)	<14.7–16.6	8.4 ± 3.1	<14.7	<14.7	<14.7	<14.7	9.2
	Ingredient	17 (4)	<14.7–17.3	7.4 ± 17.3	<14.7	<14.7	<14.7	<14.7	9.8
**AFG1**	Complete	100 (1)	<155.8	77.9	<155.8	<155.8	<155.8	<155.8	<155.8
	Complementary	0 (0)	-	-	-	-	-	-	-
	Ingredient	0 (0)	-	-	-	-	-	-	-
**AF**	Complete	43 (10)	<14.7–93.6	21.2 ± 28.1	<14.7	<14.7	<14.7	18.9	48.6
	Complementary	39 (9)	<14.7–16.6	8.4 ± 3.1	<14.7	<14.7	<14.7	<14.7	9.2
	Ingredient	17 (4)	<14.7–17.3	7.4 ± 17.3	<14.7	<14.7	<14.7	<14.7	9.8
**DON**	Complete	56 (33)	<40.4–465.1	80.2 ± 107.8	<40.4	<40.4	<40.4*	82.8	205
	Complementary	27 (16)	<40.4–819.9	273.9 ± 236.4	<40.4	<40.4	262.8	439.4	505.2
	Ingredient	17 (10)	<40.4–778.7	20.2 ± 778.7	<40.4	129.9	208.2	208.2	439.9
**DON3G**	Complete	25 (5)	<46.8–72.8	33.3 ± 22.1	<46.8	<46.8	<46.8	<46.8	53
	Complementary	45 (9)	<46.8–97.5	36.3 ± 26.8	<46.8	<46.8	<46.8	<46.8	71.7
	Ingredient	30 (6)	<46.8	23.4	<46.8	<46.8	<46.8	<46.8	<46.8
**ZEN**	Complete	32 (10)	<38.0–367.8	76.0 ± 119.2	<38.0	<38.0	<38.0	39.0	229.1
	Complementary	45 (14)	<38.0–757.9	174.3 ± 209.9	<38.0	<38.0	98.4	225.5	392.1
	Ingredient	23 (7)	<38.0–424.1	19.0 ± 424.2	30.6	48.6	111.6	111.6	181.8
**αZEL**	Complete	26 (5)	<22.2–42.7	24.6 ± 13.8	<22.2	<22.2	25.1	32.8	38.7
	Complementary	47 (9)	<22.2–288.4	82.9 ± 99.4	<22.2	<22.2	<22.2	131.5	198.2
	Ingredient	26 (5)	<22.2–158.1	11.1 ± 158.1	17.3	26.7	42.5	42.5	64.1
**βZEL**	Complete	54 (14)	<16.0–64.5	23.6 ± 19.5	<16.0	<16.0	14.7	34.7	50.2
	Complementary	35 (9)	<16.0–79.8	40.6 ± 29.3	<16.0	<16.0	39.9	64.9	77.6
	Ingredient	12 (3)	<16.0–48.7	26.9 ± 48.7	29.9	34.3	41.7	41.7	45.2
**FUMB1**	Complete	38 (16)	<63.0–311.2	100.7 ± 95.4	<63.0	<63.0	<63.0	161.8	231.3
	Complementary	40 (17)	<63.0–1427.4	324.1 ± 403.0	<63.0	<63.0	137.2	451.2	871
	Ingredient	21 (9)	<63.0–1239.3	31.5 ± 1239.3	67.7	99.3	214.3	214.3	542.6
**FUMB2**	Complete	26 (6)	<68.9–91.4	43.9 ± 23.3	<68.9	<68.9	<68.9	<68.9	62.9
	Complementary	48 (11)	<68.9–649.2	145.8 ± 185.1	<68.9	<68.9	<68.9	197.1	232.8
	Ingredient	26 (6)	<68.9–495.2	34.5 ± 495.2	<68.9	<68.9	79.7	79.7	157.5
**FUMB**	Complete	38 (16)	<63.0–345.7	117.2 ± 113.9	<63.0	<63.0	<63.0*	219.5	267.3
	Complementary	40 (17)	<63.0–2076.6	418.4 ± 553.2	<63.0	66.0	212.1	485.7	1099.4
	Ingredient	21 (9)	<63.0–1734.5	31.5 ± 1734.5	67.7	99.3	248.8	248.8	667.5
**ECO**	Complete	55 (6)	37.6–49.5	41.5 ± 4.2	38.2	39.1	40.5	42.0	45.9

Mycotoxin	Feed Type	Prevalence % (x)	Range µg/kg	Mean ± SD µg/kg	10th Percentile µg/kg	25th Percentile µg/kg	Median µg/kg	75th Percentile µg/kg	90th Percentile µg/kg
	Complementary	36 (4)	38.9–64.3	55.5 ± 11.3	45.0	54.1	59.4	60.7	62.9
	Ingredient	9 (1)	51.3	51.3	51.3	51.3	51.3	51.3	51.3
**ECR**	Complete	100 (1)	<24.9	<24.9	<24.9	<24.9	<24.9	<24.9	<24.9
	Complementary	0 (0)	-	-	-	-	-	-	-
	Ingredient	0 (0)	-	-	-	-	-	-	-
**ENV**	Complete	67 (2)	<21.9	<21.9	<21.9	<21.9	<21.9	<21.9	<21.9
	Complementary	33 (1)	<21.9	<21.9	<21.9	<21.9	<21.9	<21.9	<21.9
	Ingredient	0 (0)	-	-	-	-	-	-	-
**ESN**	Complete	80 (4)	<38.4–38.5	24.0 ± 9.7	<38.4	<38.4	<38.4	24.0	32.7
	Complementary	20 (1)	144.2	144.2	144.2	144.2	144.2	144.2	144.2
	Ingredient	0 (0)	-	-	-	-	-	-	-
**ETA**	Complete	100 (9)	<29.3–1895.6	301.5 ± 602.5	28.9	58.5	87.2	166.6	585.1
	Complementary	0 (0)	-	-	-	-	-	-	-
	Ingredient	0 (0)	-	-	-	-	-	-	-
**αECP**	Complete	100 (5)	<41.0–81.3	32.7 ± 27.2	<41.0	<41.0	<41.0	<41.0	57.0
	Complementary	0 (0)	-	-	-	-	-	-	-
	Ingredient	0 (0)	-	-	-	-	-	-	-
**ERG**	Complete	71 (15)	12.5–2055.3	217.1 ± 514.7	19.7	40.0	58.5	121.8	267.4
	Complementary	24 (5)	11.0–203.4	75.4 ± 74.6	22.1	38.9	59.5	64.3	147.8
	Ingredient	5 (1)	51.3	51.3	51.3	51.3	51.3	51.3	51.3
**FUSX**	Complete	0 (0)	-	-	-	-	-	-	-
	Complementary	67 (2)	<56.0	<56.0	<56.0	<56.0	<56.0	<56.0	<56.0
	Ingredient	33 (1)	<56.0	<56.0	<56.0	<56.0	<56.0	<56.0	<56.0
**HT2**	Complete	77 (10)	<41.6–411.8	63.5 ± 122.9	<41.6	<41.6	<41.6	<41.6	92.6
	Complementary	8 (1)	<41.6	20.8	<41.6	<41.6	<41.6	<41.6	<41.6
	Ingredient	15 (2)	<41.6–112.3	20.8 ± 112.3	30.0	43.7	66.6	66.6	89.4
**NEO**	Complete	75 (3)	<177.7	<177.7	<177.7	<177.7	<177.7	<177.7	<177.7
	Complementary	0 (0)	-	-	-	-	-	-	-
	Ingredient	25 (1)	<177.7	<177.7	<177.7	<177.7	<177.7	<177.7	<177.7
**NIV**	Complete	0 (0)	-	-	-	-	-	-	-
	Complementary	56 (5)	<40.3–69.8	48.6 ± 26.0	<40.3	<40.3	64.4	68.4	69.2
	Ingredient	44 (4)	<40.3–76.0	20.2 ± 76.0	34.0	54.8	68.8	68.8	72.5
**AOH**	Complete	67 (20)	<36.2–43.3	19.4 ± 5.6	<36.2	<36.2	<36.2	<36.2	<36.2
	Complementary	23 (7)	<36.2	<36.2	<36.2	<36.2	<36.2	<36.2	<36.2
	Ingredient	10 (3)	<36.2	<36.2	<36.2	<36.2	<36.2	<36.2	<36.2
**AME**	Complete	100 (1)	94.5	94.5	94.5	94.5	94.5	94.5	94.5
	Complementary	0 (0)	-	-	-	-	-	-	-
	Ingredient	0 (0)	-	-	-	-	-	-	-
**ENNA**	Complete	67 (2)	<26.1	<26.1	<26.1	<26.1	<26.1	<26.1	<26.1
	Complementary	33 (1)	<26.1	<26.1	<26.1	<26.1	<26.1	<26.1	<26.1
	Ingredient	0 (0)	-	-	-	-	-	-	-
**ENNA1**	Complete	40 (2)	<13.5	<13.5	<13.5	<13.5	<13.5	<13.5	<13.5
	Complementary	20 (1)	23.8	23.8	23.8	23.8	23.8	23.8	23.8
	Ingredient	40 (2)	<13.5–14.7	6.8 ± 14.7	7.5	8.7	10.7	10.7	12.7
**ENNB**	Complete	61 (43)	<38.8–136.3	49.5 ± 40.2	<38.8	<38.8	<38.8	72.2	121.9
	Complementary	25 (18)	<38.8–150.0	31.7 ± 31.7	<38.8	<38.8	<38.8	<38.8	52.0

Mycotoxin	Feed Type	Prevalence % (x)	Range µg/kg	Mean ± SD µg/kg	10th Percentile µg/kg	25th Percentile µg/kg	Median µg/kg	75th Percentile µg/kg	90th Percentile µg/kg
	Ingredient	14 (10)	<38.8–53.2	19.4 ± 53.2	<38.8	<38.8	<38.8	<38.8	36.1
**ENNB1**	Complete	44 (16)	<12.9–43.5	25.2 ± 10.0	12.3	19.1	26.6	30.8	35.7
	Complementary	39 (14)	14.4–37.7	21.1 ± 7.6	15.6	16.1	17.9	23.4	33.7
	Ingredient	17 (6)	14.6–26.7	14.6 ± 26.7	14.8	16.9	23.2	23.2	24.9
**ENN**	Complete	61 (43)	19.4–173.8	59.8 ± 47.8	19.4	19.4	42.3	88.3	132.3
	Complementary	25 (18)	19.4–186.7	50.2 ± 43.0	19.4	34.1	35.9	39.2	93.1
	Ingredient	14 (10)	19.4–94.6	19.4 ± 94.6	19.4	19.4	34.2	34.2	60.7
**CUL**	Complete	8 (1)	141.6	141.6	141.6	141.6	141.6	141.6	141.6
	Complementary	46 (6)	<42.3–187.1	121.5 ± 65.9	52.6	87.0	125.7	178	186.3
	Ingredient	46 (6)	56.1–288.7	56.1 ± 288.7	65.7	84.9	132.5	132.5	205.4
**BEA**	Complete	41 (15)	<15.9–841.8	77.0 ± 212.8	<15.9	<15.9	<15.9*	39.9	71.3
	Complementary	41 (15)	<15.9–296.7	94.0 ± 94.2	13.5	23.3	42.5	131	237.5
	Ingredient	19 (7)	<15.9–219.5	8.0 ± 219.5	22.0	32.9	69.2	69.2	98.3
**STC**	Complete	43 (3)	<30.5–162.5	64.3 ± 85.0	<30.5	<30.5	<30.5	88.9	133.1
	Complementary	57 (4)	<30.5–3517.1	986.5 ± 1696.7	<30.5	<30.5	206.8	1178	2581.5
	Ingredient	0 (0)	-	-	-	-	-	-	-
**MON**	Complete	13 (1)	2583.4	2583.4	2583.4	2583.4	2583.4	2583.4	2583.4
	Complementary	63 (5)	109.5–1181.1	497.1 ± 429.1	168.7	257.6	296.2	641.3	965.2
	Ingredient	25 (2)	419.4–1225.9	419.4 ± 1225.9	500.1	621.0	822.7	822.7	1024.3

Key: µg/kg, micrograms per kilogram; %, per cent; x, number of positive samples; AFB1, aflatoxin B1; AFG1, aflatoxin G1; AF, total aflatoxins; DON, deoxynivalenol; DON3G, deoxynivalenol-3-glucoside; ZEN, zearalenone; αZEL, alpha zearalenol; βZEL, beta zearalenol; FUMB1, fumonisin B1; FUMB2, fumonisin B2; FUMB, total fumonisins B; ECO, ergocornine; ECR, ergocristine; ENV, ergonovine; ESN, ergosine; ETA, ergotamine; αECP, alpha ergocryptine; ERG, total ergot alkaloids; FUSX, fusarenon X; HT2, HT-2 toxin; NEO, neosolaniol; NIV, nivalenol; AOH, alternariol; AME, alternariol methyl ether; ENNA, enniatin A; ENNA1, enniatin A1; ENNB, enniatin B; ENNB1, enniatin B1; ENN, total enniatins; CUL, 15 hydroxy-culmorin; BEA, beauvericin; STC, sterigmatocystin; MON, moniliformin; *, $p \leq 0.05$.

Table S2. Mycotoxins prevalence and levels in rainbow trout and tilapia feeds samples.

Mycotoxin	Fish Type	Prevalence % (x)	Range µg/kg	Mean ± SD µg/kg	10th Percentile µg/kg	25th Percentile µg/kg	Median µg/kg	75th Percentile µg/kg	90th Percentile µg/kg
**AFB1**	Rainbow trout	0 (0)	-	-	-	-	-	-	-
	Tilapia	100 (23)	<14.7–43.6	10.8 ± 8.4	<14.7	<14.7	<14.7	<14.7	17.2
**AFG1**	Rainbow trout	0 (0)	-	-	-	-	-	-	-
	Tilapia	100 (1)	<155.8	<155.8	<155.8	<155.8	<155.8	<155.8	<155.8
**AF**	Rainbow trout	0 (0)	-	-	-	-	-	-	-
	Tilapia	100 (23)	<14.7–93.6	14.2 ± 19.2	<14.7	<14.7	<14.7	<14.7	21.7
**DON**	Rainbow trout	20 (12)	<40.4–162.6	36.0 ± 42.1	<40.4	<40.4	<40.4	<40.4	62.2
	Tilapia	80 (47)	<40.4–819.9	202.8 ± 212.7	<40.4	<40.4	122.8*	353	464.3
**DON3G**	Rainbow trout	5 (1)	<46.8	<46.8	<46.8	<46.8	<46.8	<46.8	<46.8
	Tilapia	95 (19)	<46.8–97.5	32.1 ± 21.4	<46.8	<46.8	<46.8	<46.8	66.7
**ZEN**	Rainbow trout	3 (1)	<38.0	<38.0	<38.0	<38.0	<38.0	<38.0	<38.0
	Tilapia	97 (30)	<38.0–757.9	139.9 ± 172.2	<38.0	<38.0	61.8	193.4	373.3
**αZEL**	Rainbow trout	0 (0)	-	-	-	-	-	-	-
	Tilapia	100 (19)	<22.2–288.4	61.6 ± 76.1	<22.2	<22.2	26.7	79.4	161.6
**βZEL**	Rainbow trout	15 (4)	<16.0–35.6	18.3 ± 13.2	<16.0	<16.0	14.7	25	31.3
	Tilapia	85 (22)	<16.0–79.8	33.7 ± 24.4	<16.0	<16.0	30.9	49.5	64.9
**FUMB1**	Rainbow trout	7 (3)	<63.0–154.4	72.5 ± 71.0	<63.0	<63.0	<63.0	93	129.8
	Tilapia	93 (39)	<63.0–1427.4	261.1 ± 340.6	<63.0	<63.0	118.4	313.1	664.9
**FUMB2**	Rainbow trout	4 (1)	<68.9	<68.9	<68.9	<68.9	<68.9	<68.9	<68.9
	Tilapia	96 (22)	<68.9–649.2	123.9 ± 161.0	<68.9	<68.9	<68.9	157.5	231.7
**FUMB**	Rainbow trout	7 (3)	<63.0–188.9	84.0 ± 90.8	<63.0	<63.0	<63.0	110.2	157.4
	Tilapia	93 (39)	<63.0–2076.6	331 ± 467.3	<63.0	<63.0	171.7	347.6	846.2
**ECO**	Rainbow trout	0 (0)	-	-	-	-	-	-	-
	Tilapia	100 (11)	37.6–64.3	47.5 ± 9.8	38.8	39.4	42.3	55.3	59.5
**ECR**	Rainbow trout	0 (0)	-	-	-	-	-	-	-
	Tilapia	100 (1)	<24.9	<24.9	<24.9	<24.9	<24.9	<24.9	<24.9
**ENV**	Rainbow trout	0 (0)	-	-	-	-	-	-	-
	Tilapia	100 (3)	<21.9	<21.9	<21.9	<21.9	<21.9	<21.9	<21.9
**ESN**	Rainbow trout	0 (0)	-	-	-	-	-	-	-
	Tilapia	100 (5)	<38.4–144.2	48.1 ± 54.4	<38.4	<38.4	<38.4	38.5	101.9
**ETA**	Rainbow trout	0 (0)	-	-	-	-	-	-	-
	Tilapia	100 (9)	<29.3–1895.6	301.5 ± 602.5	28.9	58.5	87.2	166.6	585.1
**αECP**	Rainbow trout	20 (1)	<41.0	<41.0	<41.0	<41.0	<41.0	<41.0	<41.0
	Tilapia	80 (4)	<41.0–81.3	35.7 ± 30.4	<41.0	<41.0	<41.0	35.7	63.1
**ERG**	Rainbow trout	5 (1)	20.5	20.5	20.5	20.5	20.5	20.5	20.5
	Tilapia	95 (20)	11.0–2055.3	183.2 ± 447.1	18.5	40.6	59	116.7	216.5
**FUSX**	Rainbow trout	0 (0)	-	-	-	-	-	-	-
	Tilapia	100 (3)	<56.0	<56.0	<56.0	<56.0	<56.0	<56.0	<56.0

Mycotoxin	Fish Type	Prevalence % (x)	Range µg/kg	Mean ± SD µg/kg	10th Percentile µg/kg	25th Percentile µg/kg	Median µg/kg	75th Percentile µg/kg	90th Percentile µg/kg
**HT2**	Rainbow trout	31 (4)	<41.6–411.8	118.6 ± 195.5	<41.6	<41.6	<41.6	118.6	294.5
	Tilapia	69 (9)	<41.6–112.3	35.0 ± 31.4	<41.6	<41.6	<41.6	<41.6	68.1
**NEO**	Rainbow trout	50 (2)	<177.7	<177.7	<177.7	<177.7	<177.7	<177.7	<177.7
	Tilapia	50 (2)	<177.7	<177.7	<177.7	<177.7	<177.7	<177.7	<177.7
**NIV**	Rainbow trout	0 (0)	-	-	-	-	-	-	-
	Tilapia	100 (9)	<40.3–76	53.0 ± 24.8	<40.3	<40.3	66.3	69.8	72.2
**AOH**	Rainbow trout	3 (1)	<36.2	<36.2	<36.2	<36.2	<36.2	<36.2	<36.2
	Tilapia	97 (29)	<36.2–43.3	19.0 ± 4.7	<36.2	<36.2	<36.2	<36.2	<36.2
**AME**	Rainbow trout	0 (0)	-	-	-	-	-	-	-
	Tilapia	100 (1)	94.5	94.5	94.5	94.5	94.5	94.5	94.5
**ENNA**	Rainbow trout	0 (0)	-	-	-	-	-	-	-
	Tilapia	100 (3)	<26.1	<26.1	<26.1	<26.1	<26.1	<26.1	<26.1
**ENNA1**	Rainbow trout	0 (0)	-	-	-	-	-	-	-
	Tilapia	100 (5)	<13.5–23.8	11.8 ± 7.6	<13.5	<13.5	<13.5	14.7	20.2
**ENNB**	Rainbow trout	23 (16)	<38.8–81.4	31.5 ± 20.5	<38.8	<38.8	<38.8	40.6	63.4
	Tilapia	77 (55)	<38.8–150.0	44.9 ± 39.6	<38.8	<38.8	<38.8	57.5	121.9
**ENNB1**	Rainbow trout	11 (4)	18.7–33.9	24.5 ± 7.1	18.9	19.1	22.8	28.2	31.6
	Tilapia	89 (32)	<12.9–43.5	22.8 ± 8.8	14.6	16.3	23.2	27	36.4
**ENN**	Rainbow trout	23 (16)	19.4–115.3	37.6 ± 26.9	19.4	19.4	19.4	49	63.4
	Tilapia	77 (55)	19.4–186.7	59.9 ± 47.1	19.4	19.4	37.6	82.5	132.1
**CUL**	Rainbow trout	0 (0)	-	-	-	-	-	-	-
	Tilapia	100 (13)	<42.3–288.7	136.9 ± 73.5	59.9	84.1	141.6	185.4	216.1
**BEA**	Rainbow trout	11 (4)	<15.9–19.8	10.9 ± 5.9	<15.9	<15.9	<15.9	10.9	16.2
	Tilapia	89 (33)	<15.9–841.8	93.3 ± 154.8	<15.9	16	37.3*	112.5	218.6
**STC**	Rainbow trout	0 (0)	-	-	-	-	-	-	-
	Tilapia	100 (7)	<30.5–3517.1	591.3 ± 1298.0	<30.5	<30.5	<30.5	280.4	1645.8
**MON**	Rainbow trout	0 (0)	-	-	-	-	-	-	-
	Tilapia	100 (8)	<218.9–2583.4	839.3 ± 818.5	213.2	286.6	530.4	1192.3	1633.2

Key: µg/kg, micrograms per kilogram; %, per cent; x, number of positive samples; AFB1, aflatoxin B1; AFG1, aflatoxin G1; AF, total aflatoxins; DON, deoxynivalenol; DON3G, deoxynivalenol-3-glucoside; ZEN, zearalenone; αZEL, alpha zearalenol; βZEL, beta zearalenol; FUMB1, fumonisin B1; FUMB2, fumonisin B2; FUMB, total fumonisins B; ECO, ergocornine; ECR, ergocristine; ENV, ergonovine; ESN, ergosine; ETA, ergotamine; αECP, alpha ergocryptine; ERG, total ergot alkaloids; FUSX, fusarenon X; HT2, HT-2 toxin; NEO, neosolaniol; NIV, nivalenol; AOH, alternariol; AME, alternariol methyl ether; ENNA, enniatin A; ENNA1, enniatin A1; ENNB, enniatin B; ENNB1, enniatin B1; ENN, total enniatins; CUL, 15 hydroxy-culmorin; BEA, beauvericin; STC, sterigmatocystin; MON, moniliformin; *, $p \leq 0.05$.

Table S3. Mycotoxins prevalence and levels in fish feeds samples from fish farmers and feed manufacturers.

Mycotoxin	Feed source	Prevalence % (x)	Range µg/kg	Mean ± SD µg/kg	10th Percentile µg/kg	25th Percentile µg/kg	Median µg/kg	75th Percentile µg/kg	90th Percentile µg/kg
**AFB1**	Fish farmer	87 (20)	<14.7–22.8	9.5 ± 4.6	<14.7	<14.7	<14.7	<14.7	16.7
	Feed Manufacturer	13 (3)	<14.7–43.6	19.4 ± 20.9	<14.7	<14.7	<14.7	25.5	36.4
**AFG1**	Fish farmer	100 (1)	<155.8	<155.8	<155.8	<155.8	<155.8	<155.8	<155.8
	Feed Manufacturer	0 (0)	-	-	-	-	-	-	-
**AF**	Fish farmer	87 (20)	<14.7–93.6	13.4 ± 19.4	<14.7	<14.7	<14.7	<14.7	17.9
	Feed Manufacturer	13 (3)	<14.7–43.6	19.4 ± 20.9	<14.7	<14.7	<14.7	25.5	36.4
**DON**	Fish farmer	81 (48)	<40.4–819.9	203.0 ± 209.8	<40.4	<40.4	122.9	350.7	464.1
	Feed Manufacturer	19 (11)	<40.4	<40.4	<40.4	<40.4	<40.4*	<40.4	<40.4
**DON3G**	Fish farmer	100 (20)	<46.8–97.5	31.7 ± 20.9	<46.8	<46.8	<46.8	<46.8	66
	Feed Manufacturer	0 (0)	-	-	-	-	-	-	-
**ZEN**	Fish farmer	90 (28)	<38.0–757.9	148.5 ± 175.2	<38.0	<38.0	74.5	200.2	384.4
	Feed Manufacturer	10 (3)	<38.0	<38.0	<38.0	<38.0	<38.0	<38.0	<38.0
**αZEL**	Fish farmer	100 (19)	<22.2–288.4	61.6 ± 76.1	<22.2	<22.2	26.7	79.4	161.6
	Feed Manufacturer	0 (0)	-	-	-	-	-	-	-
**βZEL**	Fish farmer	81 (21)	<16.0–79.8	32.1 ± 23.1	<16.0	<16.0	29.8	46.6	64.9
	Feed Manufacturer	19 (5)	<16.0–64.5	28.1 ± 27.8	<16.0	<16.0	<16.0	51.8	59.4
**FUMB1**	Fish farmer	88 (37)	<63.0–1427.4	270.3 ± 346.1	<63.0	<63.0	134.4	315	703.3
	Feed Manufacturer	12 (5)	<63.0–274.4	80.1 ± 108.6	<63.0	<63.0	<63.0	<63.0	177.2
**FUMB2**	Fish farmer	96 (22)	<68.9–649.2	123.9 ± 161.0	<68.9	<68.9	<68.9	157.5	231.7
	Feed Manufacturer	4 (1)	<68.9	<68.9	<68.9	<68.9	<68.9	<68.9	<68.9
**FUMB**	Fish farmer	88 (37)	<63.0–2076.6	343.9 ± 475.3	<63.0	<63.0	188.9	349.5	897.5
	Feed Manufacturer	12 (5)	<63.0–308.9	87.0 ± 124.0	<63.0	<63.0	<63.0	<63.0	197.9
**ECO**	Fish farmer	91 (10)	37.6–64.3	48.0 ± 10.1	38.7	39.2	45.3	57.2	60
	Feed Manufacturer	9 (1)	42.3	42.3	42.3	42.3	42.3	42.3	42.3
**ECR**	Fish farmer	100 (1)	<24.9	<24.9	<24.9	<24.9	<24.9	<24.9	<24.9
	Feed Manufacturer	0 (0)	-	-	-	-	-	-	-
**ENV**	Fish farmer	67 (2)	<21.9	<21.9	<21.9	<21.9	<21.9	<21.9	<21.9
	Feed Manufacturer	33 (1)	<21.9	<21.9	<21.9	<21.9	<21.9	<21.9	<21.9
**ESN**	Fish farmer	60 (3)	<38.4–144.2	67.3 ± 67.3	23.1	28.9	38.5	91.4	123.1
	Feed Manufacturer	40 (2)	<38.4	<38.4	<38.4	<38.4	<38.4	<38.4	<38.4
**ETA**	Fish farmer	67 (6)	<29.3–1895.6	392.4 ± 738.4	36.6	65.7	109.5	157.9	1031.1
	Feed Manufacturer	33 (3)	32.4–257.5	119.8 ± 120.7	39.8	50.9	69.4	163.5	219.9
**αECP**	Fish farmer	40 (2)	<41–81.3	50.9 ± 43.0	26.6	35.7	50.9	66.1	75.2
	Feed Manufacturer	60 (3)	<41.0	<41.0	<41.0	<41.0	<41.0	<41.0	<41.0
**ERG**	Fish farmer	76 (16)	11.0–2055.3	198.3 ± 498.6	25.6	40.6	59	98.4	204.9
	Feed Manufacturer	24 (5)	19.2–308.2	102.5 ± 120.9	19.7	20.5	52.9	111.7	229.6
**FUSX**	Fish farmer	100 (3)	<56.0	<56.0	<56.0	<56.0	<56.0	<56.0	<56.0
	Feed Manufacturer	0 (0)	-	-	-	-	-	-	-

Mycotoxin	Feed source	Prevalence % (x)	Range µg/kg	Mean ± SD µg/kg	10th Percentile µg/kg	25th Percentile µg/kg	Median µg/kg	75th Percentile µg/kg	90th Percentile µg/kg
**HT2**	Fish farmer	77 (10)	<41.6–112.3	33.6 ± 29.9	<41.6	<41.6	<41.6	<41.6	62.6
	Feed Manufacturer	23 (3)	<41.6–411.8	151.1 ± 225.7	<41.6	<41.6	<41.6	216.3	333.6
**NEO**	Fish farmer	50 (2)	<177.7	<177.7	<177.7	<177.7	<177.7	<177.7	<177.7
	Feed Manufacturer	50 (2)	<177.7	<177.7	<177.7	<177.7	<177.7	<177.7	<177.7
**NIV**	Fish farmer	100 (9)	<40.3–76.0	53.0 ± 24.8	<40.3	<40.3	66.3	69.8	72.2
	Feed Manufacturer	0 (0)	-	-	-	-	-	-	-
**AOH**	Fish farmer	87 (26)	<36.2–43.3	19.1 ± 4.9	<36.2	<36.2	<36.2	<36.2	<36.2
	Feed Manufacturer	13 (4)	<36.2	<36.2	<36.2	<36.2	<36.2	<36.2	<36.2
**AME**	Fish farmer	100 (1)	94.5	94.5	94.5	94.5	94.5	94.5	94.5
	Feed Manufacturer	0 (0)	-	-	-	-	-	-	-
**ENNA**	Fish farmer	100 (3)	<26.1	<26.1	<26.1	<26.1	<26.1	<26.1	<26.1
	Feed Manufacturer	0 (0)	-	-	-	-	-	-	-
**ENNA1**	Fish farmer	80 (4)	<13.5–23.8	13.0 ± 8.1	<13.5	<13.5	10.7	17.0	21.1
	Feed Manufacturer	20 (1)	<13.5	<13.5	<13.5	<13.5	<13.5	<13.5	<13.5
**ENNB**	Fish farmer	76 (54)	<38.8–150.0	43.5 ± 38.1	<38.8	<38.8	<38.8	56.3	121.9
	Feed Manufacturer	24 (17)	6.8	6.8	6.8	6.8	6.8	6.8	6.8
**ENNB1**	Fish farmer	89 (32)	<12.9–43.5	23.1 ± 8.5	15.0	16.6	21.4	27.2	36.4
	Feed Manufacturer	11 (4)	19.4–118.4	36.7 ± 30.9	19.4	19.4	19.4	42.3	82.3
**ENN**	Fish farmer	76 (54)	19.4–186.7	58.9 ± 47.5	19.4	19.4	37.2	80.2	132.8
	Feed Manufacturer	24 (17)	<12.9–27.4	21.7 ± 10.2	12.4	21.3	26.6	27.0	27.2
**CUL**	Fish farmer	100 (13)	<42.3–288.7	136.9 ± 73.5	59.9	84.1	141.6	185.4	216.1
	Feed Manufacturer	0 (0)	19.4–118.4	42.2 ± 29.5	19.4	19.4	32.6	46.8	82.3
**BEA**	Fish farmer	81 (30)	<15.9–841.8	99.8 ± 161.0	<15.9	20.3	36.5	120.2	222.8
	Feed Manufacturer	19 (7)	<15.9	<15.9	<15.9	<15.9	<15.9*	<15.9	<15.9
**STC**	Fish farmer	86 (6)	<30.5–3517.1	662.7 ± 1406.7	<30.5	<30.5	<30.5	302.5	1957.7
	Feed Manufacturer	14 (1)	8.0–46.5	18.4 ± 17.8	8.0	8.0	8.0	25.2	44.0
**MON**	Fish farmer	100 (8)	<218.9–2583.4	839.3 ± 818.5	213.2	286.6	530.4	1192.3	1633.2
	Feed Manufacturer	0 (0)	162.5	162.5	162.5	162.5	162.5	162.5	162.5

Key: µg/kg, micrograms per kilogram; %, per cent; x, number of positive samples; AFB1, aflatoxin B1; AFG1, aflatoxin G1; AF, total aflatoxins; DON, deoxynivalenol; DON3G, deoxynivalenol-3-glucoside; ZEN, zearalenone; αZEL, alpha zearalenol; βZEL, beta zearalenol; FUMB1, fumonisin B1; FUMB2, fumonisin B2; FUMB, total fumonisins B; ECO, ergocornine; ECR, ergocristine; ENV, ergonovine; ESN, ergosine; ETA, ergotamine; αECP, alpha ergocryptine; ERG, total ergot alkaloids; FUSX, fusarenon X; HT2, HT-2 toxin; NEO, neosolaniol; NIV, nivalenol; AOH, alternariol; AME, alternariol methyl ether; ENNA, enniatin A; ENNA1, enniatin A1; ENNB, enniatin B; ENNB1, enniatin B1; ENN, total enniatins; CUL, 15 hydroxy-culmorin; BEA, beauvericin; STC, sterigmatocystin; MON, moniliformin; *, $p \leq 0.05$.

Table S4. Mycotoxins prevalence and levels in commercial and homemade fish feeds samples.

Mycotoxin	Feed preparation	Prevalence % (x)	Range μg/kg	Mean ± SD μg/kg	10th Percentile μg/kg	25th Percentile μg/kg	Median μg/kg	75th Percentile μg/kg	90th Percentile μg/kg
AFB1	Commercial	48 (11)	<14.7–43.6	13.0 ± 11.4	<14.7	<14.7	<14.7	12.3	22.8
	Homemade	52 (12)	<14.7–16.6	8.8 ± 3.4	<14.7	<14.7	<14.7	<14.7	14.9
AFG1	Commercial	0 (0)	-	-	-	-	-	-	-
	Homemade	100 (1)	<155.8	<155.8	<155.8	<155.8	<155.8	<155.8	<155.8
AF	Commercial	48 (11)	<14.7–43.6	13.0 ± 11.4	<14.7	<14.7	<14.7	12.3	22.8
	Homemade	52 (12)	<14.7–93.6	15.3 ± 24.8	<14.7	<14.7	<14.7	<14.7	15.7
DON	Commercial	69 (41)	<40.4–778.7	115.6 ± 167.6	<40.4	<40.4	<40.4*	122.9	316.7
	Homemade	31 (18)	<40.4–819.9	290.3 ± 225.1	<40.4	69.3	305.7	449.5	489.6
DON3G	Commercial	50 (10)	<46.8–72.8	28.3 ± 15.6	<46.8	<46.8	<46.8	<46.8	28.3
	Homemade	50 (10)	<46.8–97.5	35.0 ± 25.6	<46.8	<46.8	<46.8	<46.8	68.4
ZEN	Commercial	52 (16)	<38.0–757.9	156.0 ± 205.6	<38.0	<38.0	52.3	200.2	396
	Homemade	48 (15)	<38.0–423.2	114.7 ± 127.4	<38.0	<38.0	64.8	172.5	287.2
αZEL	Commercial	53 (10)	<22.2–288.4	68.9 ± 88.6	<22.2	15.0	37.7	58.8	171.1
	Homemade	47 (9)	<22.2–175.7	53.6 ± 63.7	<22.2	<22.2	<22.2	94.6	140.3
βZEL	Commercial	58 (15)	<16.0–79.8	33.6 ± 22.1	<16.0	14.7	31.9	47.7	59.4
	Homemade	42 (11)	<16.0–77.1	28.1 ± 26.1	<16.0	<16.0	<16.0	44.8	64.9
FUMB1	Commercial	52 (22)	<63.0–1239.3	195.4 ± 288.0	<63.0	<63.0	79.0	207.8	521.7
	Homemade	48 (20)	<63.0–1427.4	305.1 ± 373.4	<63.0	57.2	157.4	349.1	831.7
FUMB2	Commercial	39 (9)	<68.9–495.2	110.6 ± 152.6	<68.9	<68.9	<68.9	124.9	233.8
	Homemade	61 (14)	<68.9–649.2	126.0 ± 167.5	<68.9	<68.9	<68.9	159.7	229.5
FUMB	Commercial	52 (22)	<63.0–1734.5	240.6 ± 394.6	<63.0	<63.0	79.0	242.2	637.6
	Homemade	48 (20)	<63.0–2076.6	393.3 ± 511.6	<63.0	74.3	222.1	383.5	1063.4
ECO	Commercial	55 (6)	37.6–51.3	43.6 ± 5.5	38.8	40.2	41.7	47.7	50.4
	Homemade	45 (5)	38.8–64.3	52.1 ± 12.3	38.8	38.9	59.2	59.5	62.4
ECR	Commercial	100 (1)	<24.9	<24.9	<24.9	<24.9	<24.9	<24.9	<24.9
	Homemade	0 (0)	-	-	-	-	-	-	-
ENV	Commercial	67 (2)	<21.9	<21.9	<21.9	<21.9	<21.9	<21.9	<21.9
	Homemade	33 (1)	<21.9	<21.9	<21.9	<21.9	<21.9	<21.9	<21.9
ESN	Commercial	60 (3)	<38.4–38.5	25.6 ± 11.1	<38.4	<38.4	<38.4	28.9	34.6
	Homemade	40 (2)	<38.4–144.2	81.7 ± 88.4	31.7	50.5	81.7	113	131.7
ETA	Commercial	89 (8)	<29.3–1895.6	328.3 ± 638.3	27.1	52.0	100.6	189.3	748.9
	Homemade	11 (1)	87.2	87.2	87.2	87.2	87.2	87.2	87.2
αECP	Commercial	100 (5)	<41.0–81.3	32.7 ± 27.2	<41.0	<41.0	<41.0	<41.0	57.0
	Homemade	0 (0)	-	-	-	-	-	-	-
ERG	Commercial	62 (13)	12.5–2055.3	243.3 ± 550.7	24.6	49.5	58.5	131.8	287.8
	Homemade	38 (8)	11.0–203.4	65.3 ± 61.0	16.7	33.9	49.2	70.0	122.1
FUSX	Commercial	33 (1)	<56.0	<56.0	<56.0	<56.0	<56.0	<56.0	<56.0
	Homemade	67 (2)	<56.0	<56.0	<56.0	<56.0	<56.0	<56.0	<56.0

Mycotoxin	Feed preparation	Prevalence % (x)	Range µg/kg	Mean ± SD µg/kg	10th Percentile µg/kg	25th Percentile µg/kg	Median µg/kg	75th Percentile µg/kg	90th Percentile µg/kg
HT2	Commercial	85 (11)	<41.6–411.8	64.7 ± 118.4	<41.6	<41.6	<41.6	<41.6	112.3
	Homemade	15 (2)	<41.6–57.1	39.0 ± 25.7	24.4	29.9	39.0	48.0	53.5
NEO	Commercial	75 (3)	<177.7	<177.7	<177.7	<177.7	<177.7	<177.7	<177.7
	Homemade	25 (1)	<177.7	<177.7	<177.7	<177.7	<177.7	<177.7	<177.7
NIV	Commercial	56 (5)	<40.3–76	60.7 ± 22.9	38.6	66.3	69.8	71.3	74.1
	Homemade	44 (4)	<40.3–68.4	43.3 ± 26.8	<40.3	<40.3	42.3	65.4	67.2
AOH	Commercial	67 (20)	<36.2–43.3	19.4 ± 5.6	<36.2	<36.2	<36.2	<36.2	<36.2
	Homemade	33 (10)	<36.2	<36.2	<36.2	<36.2	<36.2	<36.2	<36.2
AME	Commercial	100 (1)	94.5	94.5	94.5	94.5	94.5	94.5	94.5
	Homemade	0 (0)	-	-	-	-	-	-	-
ENNA	Commercial	67 (2)	<26.1	<26.1	<26.1	<26.1	<26.1	<26.1	<26.1
	Homemade	33 (1)	<26.1	<26.1	<26.1	<26.1	<26.1	<26.1	<26.1
ENNA1	Commercial	60 (3)	<13.5–14.7	9.4 ± 4.6	<13.5	<13.5	<13.5	10.7	13.1
	Homemade	40 (2)	<13.5–23.8	15.3 ± 12.1	8.5	11.0	15.3	19.5	22.1
ENNB	Commercial	69 (49)	<38.8–136.3	43.0 ± 34.7	<38.8	<38.8	<38.8	61.3	90.3
	Homemade	31 (22)	<38.8–150.0	39.4 ± 40.9	<38.8	<38.8	<38.8	37.8	119.5
ENNB1	Commercial	56 (20)	<12.9–37.5	23.2 ± 7.2	14.9	18.6	23.6	27.0	30.3
	Homemade	44 (16)	<12.9–43.5	22.7 ± 10.2	15.2	16.3	19.0	28.5	37.2
ENN	Commercial	69 (49)	19.4–173.8	53.5 ± 40.0	19.4	19.4	38.6	80.8	115.9
	Homemade	31 (22)	19.4–186.7	57.9 ± 53.3	19.4	22.7	35.9	61.1	161.1
CUL	Commercial	54 (7)	56.1–288.7	150.0 ± 82.1	67.6	94.4	141.6	187.5	249.5
	Homemade	46 (6)	<42.3–187.1	121.5 ± 65.9	52.6	87.0	125.7	178.0	186.3
BEA	Commercial	57 (21)	<15.9–841.8	87.3 ± 184.0	<15.9	<15.9	31.4	69.2	214.8
	Homemade	43 (16)	<15.9–296.7	80.7 ± 87.3	<15.9	22.0	39.1	115.5	195.2
STC	Commercial	29 (2)	<30.5	<30.5	<30.5	<30.5	<30.5	<30.5	<30.5
	Homemade	71 (5)	<30.5–3517.1	821.7 ± 1514.9	<30.5	<30.5	162.5	398.3	2269.6
MON	Commercial	50 (4)	419.4–2583.4	1217.5 ± 972.1	486.0	585.8	933.6	1565.3	2176.2
	Homemade	50 (4)	<218.9–1181.1	461.1 ± 486.7	153.9	220.6	276.9	517.4	915.6

Key: µg/kg, micrograms per kilogram; %, per cent; x, number of positive samples; AFB1, aflatoxin B1; AFG1, aflatoxin G1; AF, total aflatoxins; DON, deoxynivalenol; DON3G, deoxynivalenol-3-glucoside; ZEN, zearalenone; αZEL, alpha zearalenol; βZEL, beta zearalenol; FUMB1, fumonisin B1; FUMB2, fumonisin B2; FUMB, total fumonisins B; ECO, ergocornine; ECR, ergocristine; ENV, ergonovine; ESN, ergosine; ETA, ergotamine; αECP, alpha ergocryptine; ERG, total ergot alkaloids; FUSX, fusarenon X; HT2, HT-2 toxin; NEO, neosolaniol; NIV, nivalenol; AOH, alternariol; AME, alternariol methyl ether; ENNA, enniatin A; ENNA1, enniatin A1; ENNB, enniatin B; ENNB1, enniatin B1; ENN, total enniatins; CUL, 15 hydroxy-culmorin; BEA, beauvericin; STC, sterigmatocystin; MON, moniliformin; *, $p \leq 0.05$.

Table S5. Prevalence of mycotoxins in fish feed samples containing particular ingredients.

Mycotoxin	Maize Bran	Wheat Bran	Dried Silver Cyprinid Fish	Fishmeal	Pollard	Freshwater Shrimp	Cotton Seed Cake	Sun Flower Seed Cake	Soya Bean Cake	Maize Germ	Canola Seed Cake	Cassava	Multi Vitamins	Bone Meal	Rice Bran	Dairy Meal	Wheat	Poultry Waste	Rice
	% (x)	% (x)	% (x)	% (x)	% (x)	% (x)	% (x)	% (x)	% (x)	% (x)	% (x)	% (x)	% (x)	% (x)	% (x)	% (x)	% (x)	% (x)	% (x)
All Samples	53 (41)	47 (37)	35 (27)	35 (27)	32 (25)	28 (22)	27 (21)	27 (21)	26 (20)	14 (11)	8 (6)	8 (6)	6 (5)	5 (4)	4 (3)	2 (2)	3 (2)	3 (2)	1 (1)
AFB1	57 (13)	43 (10)	26 (6)	35 (8)	13 (3)	13 (3)	30 (7)	30 (7)	30 (7)	0 (0)	0 (0)	9 (2)	13 (3)	13 (3)	0 (0)	9 (2)	0 (0)	0 (0)	0 (0)
AFG1	100 (1)	0 (0)	0 (0)	100 (1)	0 (0)	0 (0)	0 (0)	100 (1)	100 (1)	0 (0)	0 (0)	0 (0)	0 (0)	0 (0)	0 (0)	0 (0)	0 (0)	0 (0)	0 (0)
AF	57 (13)	43 (10)	26 (6)	35 (8)	13 (3)	13 (3)	30 (7)	30 (7)	30 (7)	0 (0)	0 (0)	9 (2)	13 (3)	13 (3)	0 (0)	0 (0)	0 (0)	0 (0)	0 (0)
DON	47 (28)	44 (26)	27 (16)	29 (17)	24 (14)	19 (11)	22 (13)	22 (13)	20 (12)	14 (8)	8 (5)	2 (1)	8 (5)	7 (4)	5 (3)	2 (1)	2 (1)	0 (0)	2 (1)
DON3G	45 (9)	35 (7)	10 (2)	20 (4)	15 (3)	5 (1)	15 (3)	15 (3)	15 (3)	0 (0)	0 (0)	5 (1)	5 (1)	5 (1)	10 (2)	5 (1)	0 (0)	0 (0)	5 (1)
ZEN	48 (15)	42 (13)	19 (6)	23 (7)	23 (7)	19 (6)	19 (6)	23 (7)	19 (6)	0 (0)	0 (0)	10 (3)	6 (2)	3 (1)	10 (3)	3 (1)	3 (1)	6 (2)	3 (1)
αZEL	42 (8)	42 (8)	11 (2)	16 (3)	5 (1)	5 (1)	11 (2)	16 (3)	11 (2)	0 (0)	0 (0)	0 (0)	11 (2)	5 (1)	5 (1)	5 (1)	5 (1)	5 (1)	5 (1)
βZEL	50 (13)	42 (11)	31 (8)	31 (8)	19 (5)	19 (5)	15 (4)	19 (5)	19 (5)	12 (3)	0 (0)	4 (1)	15 (4)	12 (3)	4 (1)	0 (0)	0 (0)	8 (2)	0 (0)
FUMB1	50 (21)	43 (18)	19 (8)	24 (10)	17 (7)	14 (6)	19 (8)	19 (8)	17 (7)	5 (2)	2 (1)	5 (2)	10 (4)	7 (3)	7 (3)	2 (1)	2 (1)	2 (1)	2 (1)
FUMB2	52 (12)	35 (8)	13 (3)	17 (4)	9 (2)	9 (2)	9 (2)	13 (3)	13 (3)	0 (0)	0 (0)	4 (1)	9 (2)	4 (1)	13 (3)	0 (0)	0 (0)	4 (1)	0 (0)
FUMB	50 (21)	43 (18)	19 (8)	24 (10)	17 (7)	14 (6)	19 (8)	19 (8)	17 (7)	5 (2)	2 (1)	5 (2)	10 (4)	7 (3)	7 (3)	2 (1)	2 (1)	2 (1)	2 (1)
ECO	45 (5)	36 (4)	9 (1)	9 (1)	9 (1)	9 (1)	9 (1)	18 (2)	9 (1)	0 (0)	0 (0)	0 (0)	9 (1)	9 (1)	0 (0)	0 (0)	0 (0)	0 (0)	0 (0)
ECR	0 (0)	0 (0)	0 (0)	0 (0)	0 (0)	0 (0)	0 (0)	0 (0)	0 (0)	0 (0)	0 (0)	0 (0)	0 (0)	0 (0)	0 (0)	0 (0)	0 (0)	0 (0)	0 (0)
ENV	33 (1)	67 (2)	33 (1)	33 (1)	0 (0)	0 (0)	33 (1)	33 (1)	33 (1)	0 (0)	0 (0)	0 (0)	33 (1)	33 (1)	0 (0)	0 (0)	0 (0)	0 (0)	0 (0)
ESN	40 (2)	60 (3)	40 (2)	40 (2)	20 (1)	20 (1)	40 (2)	40 (2)	40 (2)	0 (0)	0 (0)	20 (1)	20 (1)	20 (1)	0 (0)	0 (0)	0 (0)	0 (0)	0 (0)
ETA	33 (3)	33 (3)	33 (3)	33 (3)	0 (0)	0 (0)	33 (3)	33 (3)	33 (3)	0 (0)	0 (0)	0 (0)	33 (3)	33 (3)	0 (0)	0 (0)	0 (0)	0 (0)	0 (0)
αECP	60 (3)	60 (3)	60 (3)	60 (3)	20 (1)	20 (1)	60 (3)	60 (3)	60 (3)	20 (1)	20 (1)	0 (0)	40 (2)	40 (2)	0 (0)	0 (0)	0 (0)	0 (0)	0 (0)
ERG	43 (9)	43 (9)	24 (5)	24 (5)	14 (3)	14 (3)	24 (5)	29 (6)	24 (5)	5 (1)	5 (1)	5 (1)	14 (3)	14 (3)	0 (0)	0 (0)	0 (0)	0 (0)	0 (0)
FUSX	67 (2)	33 (1)	33 (1)	33 (1)	33 (1)	0 (0)	33 (1)	33 (1)	33 (1)	0 (0)	0 (0)	0 (0)	33 (1)	33 (1)	0 (0)	0 (0)	0 (0)	0 (0)	0 (0)
HT2	54 (7)	62 (8)	46 (6)	38 (5)	38 (5)	31 (4)	38 (5)	38 (5)	31 (4)	15 (2)	8 (1)	8 (1)	15 (2)	15 (2)	0 (0)	8 (1)	0 (0)	0 (0)	8 (1)
NEO	75 (3)	75 (3)	50 (2)	75 (3)	75 (3)	50 (2)	75 (3)	75 (3)	75 (3)	50 (2)	50 (2)	0 (0)	0 (0)	0 (0)	0 (0)	25 (1)	0 (0)	0 (0)	25 (1)
NIV	44 (4)	44 (4)	22 (2)	11 (1)	22 (2)	0 (0)	22 (2)	11 (1)	11 (1)	0 (0)	0 (0)	0 (0)	22 (2)	11 (1)	11 (1)	11 (1)	11 (1)	0 (0)	11 (1)
AOH	40 (12)	43 (13)	27 (8)	27 (8)	13 (4)	10 (3)	27 (8)	27 (8)	23 (7)	0 (0)	0 (0)	3 (1)	17 (5)	13 (4)	3 (1)	3 (1)	0 (0)	0 (0)	3 (1)

AME	100 (1)	100 (1)	100 (1)	100 (1)	100 (1)	100 (1)	100 (1)	100 (1)	100 (1)	0 (0)	0 (0)	100 (1)	0 (0)	0 (0)	0 (0)	0 (0)	0 (0)	0 (0)	0 (0)
ENNA	0 (0)	0 (0)	0 (0)	0 (0)	33 (1)	0 (0)	0 (0)	0 (0)	0 (0)	0 (0)	0 (0)	0 (0)	0 (0)	0 (0)	0 (0)	0 (0)	0 (0)	0 (0)	0 (0)
ENNA1	60 (3)	60 (3)	40 (2)	40 (2)	60 (3)	40 (2)	40 (2)	40 (2)	40 (2)	0 (0)	0 (0)	40 (2)	0 (0)	0 (0)	0 (0)	0 (0)	0 (0)	20 (1)	0 (0)
ENNB	51 (36)	46 (33)	32 (23)	32 (23)	30 (21)	25 (18)	25 (18)	25 (18)	24 (17)	14 (10)	8 (6)	4 (3)	8 (6)	6 (4)	4 (3)	1 (1)	3 (2)	3 (2)	1 (1)
ENNB1	47 (17)	44 (16)	19 (7)	25 (9)	22 (8)	17 (6)	19 (7)	14 (5)	14 (5)	6 (2)	0 (0)	6 (2)	6 (2)	6 (2)	0 (0)	3 (1)	3 (1)	6 (2)	3 (1)
ENN	51 (36)	46 (33)	32 (23)	32 (23)	30 (21)	25 (18)	25 (18)	25 (18)	24 (17)	14 (10)	8 (6)	4 (3)	8 (6)	6 (4)	4 (3)	1 (1)	3 (2)	3 (2)	1 (1)
CUL	46 (6)	46 (6)	15 (2)	8 (1)	15 (2)	8 (1)	15 (2)	8 (1)	8 (1)	0 (0)	0 (0)	0 (0)	15 (2)	8 (1)	15 (2)	8 (1)	0 (0)	15 (2)	8 (1)
BEA	54 (20)	49 (18)	27 (10)	32 (12)	24 (9)	24 (9)	30 (11)	30 (11)	27 (10)	5 (2)	5 (2)	11 (4)	11 (4)	5 (2)	8 (3)	3 (1)	3 (1)	5 (2)	3 (1)
STC	14 (1)	29 (2)	14 (1)	14 (1)	14 (1)	14 (1)	14 (1)	14 (1)	14 (1)	0 (0)	0 (0)	14 (1)	0 (0)	0 (0)	0 (0)	0 (0)	0 (0)	0 (0)	0 (0)
MON	50 (4)	25 (2)	25 (2)	13 (1)	0 (0)	0 (0)	13 (1)	13 (1)	13 (1)	0 (0)	0 (0)	0 (0)	25 (2)	13 (1)	25 (2)	0 (0)	13 (1)	0 (0)	0 (0)

Key: %, per cent; x, number of positive samples; AFB1, aflatoxin B1; AFG1, aflatoxin G1; AF, total aflatoxins; DON, deoxynivalenol; DON3G, deoxynivalenol-3-glucoside; ZEN, zearalenone; αZEL, alpha zearalenol; βZEL, beta zearalenol; FUMB1, fumonisin B1; FUMB2, fumonisin B2; FUMB, total fumonisins B; ECO, ergocornine; ECR, ergocristine; ENV, ergonovine; ESN, ergosine; ETA, ergotamine; αECP, alpha ergocryptine; ERG, total ergot alkaloids; FUSX, fusarenon X; HT2, HT-2 toxin; NEO, neosolaniol; NIV, nivalenol; AOH, alternariol; AME, alternariol methyl ether; ENNA, enniatin A; ENNA1, enniatin A1; ENNB, enniatin B; ENNB1, enniatin B1; ENN, total enniatins; CUL, 15 hydroxy-culmorin; BEA, beauvericin; STC, sterigmatocystin; MON, moniliformin.

Table S6. Median mycotoxin levels in fish feed samples containing particular ingredient.

Mycotoxin	Ingredi ent was	Maize bran	Wheat bran	Dried silver cyprini d fish	Fish meal	Pollard	Fresh water shrimp	Cotton seed cake	Sun flower seed cake	Soya bean cake	Maize germ	Canola seed cake	Cassav a	Multi vitami ns	Bone meal	Rice bran	Dairy meal	Wheat	Poultr y waste	Rice
		µg/kg	µg/kg	µg/kg	µg/kg	µg/kg	µg/kg	µg/kg	µg/kg	µg/kg	µg/kg	µg/kg	µg/kg	µg/kg	µg/kg	µg/kg	µg/kg	µg/kg	µg/kg	µg/kg
AFB1	Present	<14.7	<14.7	<14.7	<14.7	<14.7	<14.7	<14.7	<14.7	<14.7	-	-	<14.7	<14.7	<14.7	-	<14.7	-	-	-
	Absent	<14.7	<14.7	<14.7	<14.7	<14.7	<14.7	<14.7	<14.7	<14.7	<14.7	<14.7	<14.7	<14.7	<14.7	<14.7	<14.7	<14.7	<14.7	<14.7
AFG1	Present	<155.8	-	-	<155.8	-	-	-	<155.8	<155.8	-	-	-	-	-	-	-	-	-	-
	Absent		<155.8	<155.8	-	<155.8	<155.8	<155.8	-	-	<155.8	<155.8	<155.8	<155.8	<155.8	<155.8	<155.8	<155.8	<155.8	<155.8
AF	Present	<14.7	<14.7	<14.7	<14.7	<14.7	<14.7	<14.7	<14.7	<14.7	-	-	<14.7	<14.7	<14.7	-	<14.7	-	-	-
	Absent	<14.7	<14.7	<14.7	<14.7	<14.7	<14.7	<14.7	<14.7	<14.7	<14.7	<14.7	<14.7	<14.7	<14.7	<14.7	<14.7	<14.7	<14.7	<14.7
DON	Present	121.3	<40.4	<40.4*	<40.4*	<40.4	<40.4*	<40.4	<40.4	<40.4	<40.4	<40.4	316.7	<40.4	<40.4	546.7	<40.4	262.9	-	778.7
	Absent	65.6	82.5	113.5	98.5	83.4	83.1	98.5	83.1	83.4	83.4	83.1	66.3	74.7	82.5	65.4	82.7	66.3	66.9	66.3
DON3G	Present	<46.8	<46.8	<46.8	<46.8	<46.8	<46.8	<46.8	<46.8	<46.8	-	-	<46.8	<46.8	<46.8	<46.8	<46.8	-	-	<46.8
	Absent	<46.8	<46.8	<46.8	<46.8	<46.8	<46.8	<46.8	<46.8	<46.8	<46.8	<46.8	<46.8	<46.8	<46.8	<46.8	<46.8	<46.8	<46.8	<46.8
ZEN	Present	<38	58.8	116.4	<38	<38	<38	38.9	<38	<38	-	-	<38	331.3	238.5	38.4	319.6	186.5	140.3	58.8
	Absent	61.8	78.7	58.8	74.5	74.5	64.8	64.8	74.5	64.8	58.8	58.8	61.8	45.7	52.3	61.8	52.3	52.3	45.7	55.3
αZEL	Present	18.1	18.9	126.4	25.1	26.7	<22.2	60.7	25.1	59.9	-	-	-	126.4	94.6	158.1	149.8	131.5	<22.2	26.7
	Absent	32.8	32.8	25.1	29.8	29.0	29.8	25.1	29.8	26.7	26.7	26.7	26.7	25.1	25.9	25.9	26.7	25.9	29.8	29.0
βZEL	Present	<16	21.4	25.6	14.7	<16	<16	40.8	29.8	29.8	<16	-	<16	50.3	51.8	48.7	78.5	-	17.5	-
	Absent	35.6	31.9	29.4	33.8	31.9	35.6	25.1	26.9	26.9	31.9	28.4	29.8	22.3	23.2	26.9	25.1	28.4	30.9	28.4
FUMB1	Present	115.2	71.4	<63	48.6	81.2	56.4	54.3	<63	<63	<63	<63	177.8	<63	<63	333.7	<63	118.4	232.4	99.3
	Absent	134.4	145.8	135.8	145.8	134.4	126.4	145.8	135.8	134.4	126.4	118.4	116.8	126.4	134.4	99.3	134.4	115.2	115.2	118.4
FUMB2	Present	<68.9	123.2	<68.9	<68.9	<68.9	<68.9	<68.9	<68.9	<68.9	-	-	<68.9	<68.9	<68.9	<68.9	-	-	<68.9	-
	Absent	<68.9	<68.9	<68.9	<68.9	<68.9	<68.9	<68.9	<68.9	<68.9	<68.9	<68.9	<68.9	<68.9	<68.9	<68.9	<68.9	<68.9	<68.9	<68.9
FUMB	Present	149.7	79.2	48.7	71.5	81.2	56.4	71.5	48.7	66.0	<63	<63	195.0	48.7	<63	368.2	<63	118.4	266.9	99.3
	Absent	188.9	200.5	200.5	200.5	212.1	180.3	215.3	200.5	188.9	180.3	171.7	160.7	180.3	188.9	118.4	188.9	171.7	149.7	171.7
ECO	Present	49.5	45.9	42.3	42.3	59.5	49.5	42.3	45.9	42.3	-	-	-	42.3	42.3	-	64.3	-	-	-
	Absent	40.5	41.1	45.3	45.3	41.7	41.7	45.3	41.1	45.3	42.3	42.3	42.3	45.3	45.3	42.3	41.7	42.3	42.3	42.3
ECR	Present	-	-	-	-	-	-	-	-	-	-	-	-	-	-	-	-	-	-	-
	Absent	<24.9	<24.9	<24.9	<24.9	<24.9	<24.9	<24.9	<24.9	<24.9	<24.9	<24.9	<24.9	<24.9	<24.9	<24.9	<24.9	<24.9	<24.9	<24.9

**Mycotoxin**	**Ingredient was**	**Maize bran**	**Wheat bran**	**Dried silver cyprinid fish**	**Fish meal**	**Pollard**	**Fresh water shrimp**	**Cotton seed cake**	**Sun flower seed cake**	**Soya bean cake**	**Maize germ**	**Canola seed cake**	**Cassava**	**Multi vitamins**	**Bone meal**	**Rice bran**	**Dairy meal**	**Wheat**	**Poultry waste**	**Rice**
		**µg/kg**	**µg/kg**	**µg/kg**	**µg/kg**	**µg/kg**	**µg/kg**	**µg/kg**	**µg/kg**	**µg/kg**	**µg/kg**	**µg/kg**	**µg/kg**	**µg/kg**	**µg/kg**	**µg/kg**	**µg/kg**	**µg/kg**	**µg/kg**	**µg/kg**
ENV	Present	<21.9	<21.9	<21.9	<21.9	-	-	<21.9	<21.9	<21.9	-	-	-	<21.9	<21.9	-	-	-	-	-
	Absent	<21.9	<21.9	<21.9	<21.9	<21.9	<21.9	<21.9	<21.9	<21.9	<21.9	<21.9	<21.9	<21.9	<21.9	<21.9	<21.9	<21.9	<21.9	<21.9
ESN	Present	<38.4	<38.4	<38.4	<38.4	<38.4	<38.4	<38.4	<38.4	<38.4	-	-	<38.4	<38.4	<38.4	-	-	-	-	-
	Absent	38.5	28.9	38.5	38.5	28.9	28.9	38.5	38.5	38.5	<38.4	<38.4	28.9	28.9	28.9	<38.4	<38.4	<38.4	<38.4	<38.4
ETA	Present	69.4	69.4	69.4	69.4	-	-	69.4	69.4	69.4	-	-	-	69.4	69.4	-	-	-	-	-
	Absent	109.5	109.5	109.5	109.5	87.2	87.2	109.5	109.5	109.5	87.2	87.2	87.2	109.5	109.5	87.2	87.2	87.2	87.2	87.2
αECP	Present	<41	<41	<41	<41	<41	<41	<41	<41	<41	<41	<41	-	<41	<41	-	-	-	-	-
	Absent	50.9	50.9	50.9	50.9	<41	<41	50.9	50.9	50.9	<41	<41	<41	<41	<41	<41	<41	<41	<41	<41
ERG	Present	51.3	49.5	52.9	52.9	20.5	20.5	52.9	51.2	52.9	20.5	20.5	19.2	111.7	111.7	-	64.3	-	-	-
	Absent	63.8	61.4	59.0	59.0	60.9	61.4	59.0	59.5	59.0	59.0	59.0	59.0	54.9	54.9	58.5	55.7	58.5	58.5	58.5
FUSX	Present	<56	<56	<56	<56	<56	-	<56	<56	<56	-	-	-	<56	<56	-	-	-	-	-
	Absent	<56	<56	<56	<56	<56	<56	<56	<56	<56	<56	<56	<56	<56	<56	<56	<56	<56	<56	<56
HT2	Present	<41.6	<41.6	<41.6	<41.6	<41.6	<41.6	<41.6	<41.6	<41.6	216.3	<41.6	<41.6	<41.6	<41.6	-	-	-	-	<41.6
	Absent	<41.6	<41.6	<41.6	<41.6	<41.6	<41.6	<41.6	<41.6	<41.6	<41.6	<41.6	<41.6	<41.6	<41.6	<41.6	<41.6	<41.6	<41.6	<41.6
NEO	Present	<177.7	<177.7	<177.7	<177.7	<177.7	<177.7	<177.7	<177.7	<177.7	<177.7	<177.7	-	-	-	-	-	-	-	<177.7
	Absent	<177.7	<177.7	<177.7	<177.7	<177.7	<177.7	<177.7	<177.7	<177.7	<177.7	<177.7	<177.7	<177.7	<177.7	<177.7	<177.7	<177.7	<177.7	<177.7
NIV	Present	<40.3	67.4	44.3	68.4	43.2	-	67.4	68.4	68.4	-	-	-	44.3	68.4	<40.3	67.1	<40.3	-	66.3
	Absent	69.8	64.4	66.3	65.4	68.4	66.3	64.4	65.4	65.4	66.3	66.3	66.3	66.3	65.4	67.4	66.3	67.4	66.3	66.4
AOH	Present	<36.2	<36.2	<36.2	<36.2	<36.2	<36.2	<36.2	<36.2	<36.2	-	-	43.3	<36.2	<36.2	<36.2	<36.2	-	-	<36.2
	Absent	<36.2	<36.2	<36.2	<36.2	<36.2	<36.2	<36.2	<36.2	<36.2	<36.2	<36.2	<36.2	<36.2	<36.2	<36.2	<36.2	<36.2	<36.2	<36.2
AME	Present	94.5	94.5	94.5	94.5	94.5	94.5	94.5	94.5	94.5	-	-	94.5	-	-	-	-	-	-	-
	Absent	-	-	-	-	-	-	-	-	-	94.5	94.5	-	94.5	94.5	94.5	94.5	94.5	94.5	94.5
ENNA	Present	-	-	-	-	<26.1	-	-	-	-	-	-	-	-	-	-	<26.1	-	-	-
	Absent	<26.1	<26.1	<26.1	<26.1	<26.1	<26.1	<26.1	<26.1	<26.1	<26.1	<26.1	<26.1	<26.1	<26.1	<26.1	<26.1	<26.1	<26.1	<26.1
ENNA1	Present	<13.5	<13.5	<13.5	<13.5	<13.5	<13.5	<13.5	<13.5	<13.5	-	-	<13.5	-	-	-	23.8	-	<13.5	-
	Absent	15.3	15.3	14.7	14.7	10.7	14.7	14.7	14.7	14.7	<13.5	<13.5	14.7	<13.5	<13.5	<13.5	<13.5	<13.5	10.7	<13.5
ENNB	Present	<38.8	<38.8	<38.8	<38.8	<38.8	<38.8	<38.8	<38.8	<38.8	<38.8	<38.8	<38.8	<38.8	51.4	<38.8	<38.8	<38.8	<38.8	41.6

**Mycotoxin**	**Ingredient was**	**Maize bran**	**Wheat bran**	**Dried silver cyprinid fish**	**Fish meal**	**Pollard**	**Fresh water shrimp**	**Cotton seed cake**	**Sun flower seed cake**	**Soya bean cake**	**Maize germ**	**Canola seed cake**	**Cassava**	**Multi vitamins**	**Bone meal**	**Rice bran**	**Dairy meal**	**Wheat**	**Poultry waste**	**Rice**
		**μg/kg**	**μg/kg**	**μg/kg**	**μg/kg**	**μg/kg**	**μg/kg**	**μg/kg**	**μg/kg**	**μg/kg**	**μg/kg**	**μg/kg**	**μg/kg**	**μg/kg**	**μg/kg**	**μg/kg**	**μg/kg**	**μg/kg**	**μg/kg**	**μg/kg**
	Absent	<38.8	<38.8	<38.8	<38.8	<38.8	<38.8	<38.8	<38.8	<38.8	<38.8	<38.8	<38.8	<38.8	<38.8	<38.8	<38.8	<38.8	<38.8	<38.8
ENNB1	Present	19.6	25.4	26.8	26.3	26.6	26.6	22.8	26.8	26.8	30.1	-	18.2	22.9	22.9	-	16.0	19.6	19.8	22.8
	Absent	23.5	19.4	19.9	22.8	19.8	21.4	23.5	22.8	22.8	21.4	23.2	23.2	23.2	23.2	23.2	23.5	23.5	23.2	23.5
ENN	Present	35.6	37.8	32.6	37.8	32.6	26.0	40.1	35.2	37.8	19.4	19.4	32.6	42.3	65.1	19.4	35.4	29.2	42.5	64.4
	Absent	49.8	36.1	37.2	36.3	37.7	37.8	35.8	36.8	36.4	37.6	37.6	37.2	36.0	35.8	37.7	37.2	36.8	36.8	36.4
CUL	Present	153.7	132.5	117.8	84.1	149.5	155.9	98.8	84.1	84.1	-	-	-	117.8	84.1	113.4	185.4	-	222.3	113.5
	Absent	113.5	141.6	141.6	146.6	141.6	127.6	151.5	146.6	146.6	141.6	141.6	141.6	141.6	146.6	141.6	127.6	141.6	113.5	146.6
BEA	Present	28.2	35.0	21.2	<15.9	31.4	16.0	<15.9	16.0	22.6	<15.9	<15.9	39.9	21.2	152.3	34.4	31.4	137.7	54.4	69.2
	Absent	35.6	31.4	35.6	35.6	35.0	35.0	35.0	35.0	34.4	35.6	35.6	31.4	35.6	34.4	33.5	35.0	32.9	34.4	32.9
STC	Present	162.5	1839.8	162.5	162.5	162.5	162.5	162.5	162.5	162.5	-	-	162.5	-	-	-	<30.5	-	-	-
	Absent	<30.5	<30.5	<30.5	<30.5	<30.5	<30.5	<30.5	<30.5	<30.5	<30.5	<30.5	<30.5	<30.5	<30.5	<30.5	162.5	<30.5	<30.5	<30.5
MON	Present	719.4	741.8	741.8	257.6	-	-	257.6	257.6	257.6	-	-	-	741.8	257.6	761.1	641.3	<218.9	-	-
	Absent	530.4	530.4	530.4	641.3	530.4	530.4	641.3	641.3	641.3	530.4	530.4	530.4	530.4	641.3	530.4	419.4	641.3	530.4	530.4

Key: %, per cent; AFB1, aflatoxin B1; AFG1, aflatoxin G1; AF, total aflatoxins; DON, deoxynivalenol; DON3G, deoxynivalenol-3-glucoside; ZEN, zearalenone; αZEL, alpha zearalenol; βZEL, beta zearalenol; FUMB1, fumonisin B1; FUMB2, fumonisin B2; FUMB, total fumonisins B; ECO, ergocornine; ECR, ergocristine; ENV, ergonovine; ESN, ergosine; ETA, ergotamine; αECP, alpha ergocryptine; ERG, total ergot alkaloids; FUSX, fusarenon X; HT2, HT-2 toxin; NEO, neosolaniol; NIV, nivalenol; AOH, alternariol; AME, alternariol methyl ether; ENNA, enniatin A; ENNA1, enniatin A1; ENNB, enniatin B; ENNB1, enniatin B1; ENN, total enniatins; CUL, 15 hydroxy-culmorin; BEA, beauvericin; STC, sterigmatocystin; MON, moniliformin; *, $p \leq 0.05$.

Table S7. Performance parameters of the multi-mycotoxin HPLC-HRMS method used.

Mycotoxins	LOD µg/kg	LOQ µg/kg	r^2	Recovery %	SSE %
Aflatoxins					
AFB1	14.7	49.1	0.9999	79.8	87.1
AFG1	155.8	519.3	0.9995	51.6	84.6
AFB2	18.1	60.4	0.9999	78.8	82.4
AFG2	152.9	509.7	0.9994	44.8	88.9
Deoxynivalenol and its analogues					
DON	40.4	134.8	0.9991	107.1	195.7
DON3G	46.8	156.1	0.9993	58.2	252.4
3ADON	36.0	119.9	1.0000	87.7	77.7
15ADON	122.7	409	0.9993	119.8	35.5
Zearalenone and its analogues					
ZEN	38.0	126.6	0.9995	119.1	78.2
αZEL	22.2	73.9	0.9995	132.6	68.4
βZEL	16.0	53.3	0.9994	133.8	69.7
Fumonisins					
FUMB1	63.0	209.9	0.9977	52.5	61.4
FUMB2	68.9	229.7	0.9995	57.2	68.1
Ergot alkaloids					
ECO	20.7	69.1	0.9993	129.7	172.6
ECR	24.9	83.0	0.9990	142.6	227.1
ENV	21.9	73.1	0.9999	84.2	49.6
ESN	38.4	128.2	0.9996	108.8	132.7
ETA	29.3	97.6	0.9987	122.1	160.8
αECP	41.0	136.6	0.9988	136.1	231.7
Other trichothecenes					
FUSX	56.0	186.6	0.9997	106.3	114.5
HT2	41.6	138.7	0.9992	119.7	89.1
T2	58.2	194.1	1.0000	120.2	103.2
T2 triol	122.7	409.0	0.9975	121.9	108.9
T2 tetraol	41.9	139.8	0.9998	78.5	100.0
NEO	177.7	592.4	0.9999	139.8	81.0
NIV	40.3	134.2	0.9995	43.7	285.4
DAS	33.9	113.1	0.9994	118.9	78.4
Alternariol and its analogues					
AOH	36.2	120.8	0.9953	81.6	59.3
AME	16.4	54.7	0.9953	81.6	66.7
Enniatins					
ENNA	26.1	87.2	0.9980	138	263.2

**Mycotoxins**	**LOD µg/kg**	**LOQ µg/kg**	r^2	**Recovery %**	**SSE %**
ENNA1	13.5	44.9	0.9988	141.3	282.4
ENNB	38.8	129.4	0.9993	128.6	165.9
ENNB1	12.9	43.1	0.9992	134.6	211.6
Other mycotoxins					
CUL	42.3	141.0	0.9998	120.3	83.4
BEA	15.9	53.2	0.9990	133.5	202.4
STC	30.5	101.6	0.9994	127.3	116.0
MON	218.9	729.6	0.9891	71.8	100.0
BUT	149.2	497.3	0.9998	145.4	100.0
AOD	30.9	103.0	0.9891	78.9	183.9
OTA	27.3	91.1	0.9999	103.4	123.4

Key: LOD, Limit of detection; LOQ, Limit of quantification; <, less than; r^2, calibration curve regression coefficient; SSE, Signal Suppression or Enhancement; µg/kg, micrograms per kilogram; %, per cent; AFB1, aflatoxin B1; AFG1, aflatoxin G1; AFB2, aflatoxin B2; AFG2, aflatoxin G2; DON, deoxynivalenol; DON3G, deoxynivalenol 3-glucoside; 3ADON, 3-acetyldeoxynivalenol; 15ADON, 15-acetyldeoxynivalenol; ZEN, zearalenone; αZEL, alpha zearalenol; βZEL, beta zearalenol; FUMB1, fumonisin B1; FUMB2, fumonisin B2; ECO, ergocornine; ECR, ergocristine; ENV, ergonovine; ESN, ergosine; ETA, ergotamine; αECP, alpha ergocryptine; FUSX, fusarenon X; HT2, HT-2 toxin; T2, T-2 toxin; NEO, neosolaniol; NIV, nivalenol; DAS, diacetoxyscirpenol; AOH, alternariol; AME, alternariol methyl ether; ENNA, enniatin A; ENNA1, enniatin A1; ENNB, enniatin B; ENNB1, enniatin B1; CUL, 15-hydroxy-culmorin; BEA, beauvericin; STC, sterigmatocystin; MON, moniliformin; BUT, 5-acetamido-butenolide; AOD, 2-amino-14,16-dimethyloctadecan-3-ol; OTA, ochratoxin.
